# Enhanced axonal response of mitochondria to demyelination offers neuroprotection: implications for multiple sclerosis

**DOI:** 10.1007/s00401-020-02179-x

**Published:** 2020-06-22

**Authors:** Simon Licht-Mayer, Graham R. Campbell, Marco Canizares, Arpan R. Mehta, Angus B. Gane, Katie McGill, Aniket Ghosh, Alexander Fullerton, Niels Menezes, Jasmine Dean, Jordon Dunham, Sarah Al-Azki, Gareth Pryce, Stephanie Zandee, Chao Zhao, Markus Kipp, Kenneth J. Smith, David Baker, Daniel Altmann, Stephen M. Anderton, Yolanda S. Kap, Jon D. Laman, Bert A.‘t Hart, Moses Rodriguez, Ralf Watzlawick, Jan M. Schwab, Roderick Carter, Nicholas Morton, Michele Zagnoni, Robin J. M. Franklin, Rory Mitchell, Sue Fleetwood-Walker, David A. Lyons, Siddharthan Chandran, Hans Lassmann, Bruce D. Trapp, Don J. Mahad

**Affiliations:** 1 Centre for Clinical Brain Sciences, University of Edinburgh, Chancellor’s Building, 49 Little France Crescent, Edinburgh, EH16 4SB UK; 2 UK Dementia Research Institute, University of Edinburgh, Edinburgh, UK; 3 Department of Neuroscience, Lerner Research Institute, Cleveland Clinic, Cleveland, OH OH44195 USA; 4 Barts and The London School of Medicine and Dentistry, Blizard Institute, Queen Mary University of London, 4 Newark Street, London, E1 2AT UK; 5 Centre for Inflammation Research, University of Edinburgh, 47 Little France Crescent, Edinburgh, EH16 4SB UK; 6 Wellcome Trust-MRC Cambridge Stem Cell Institute, Jeffrey Cheah Biomedical Centre, University of Cambridge, Cambridge Biomedical Campus, Cambridge, CB2 0AW UK; 7 Institute of Anatomy, Rostock University Medical Center, Gertrudenstrasse 9, 18057 Rostock, Germany; 8 Department of Neuroinflammation, The UCL Queen Square Institute of Neurology, University College London, 1 Wakefield Street, London, WC1N 1PJ UK; 9 Faculty of Medicine, Department of Medicine, Hammersmith Campus, London, UK; 10 Department of Immunobiology, Biomedical Primate Research Centre, Rijswijk, The Netherlands; 11 Dept. Biomedical Sciences of Cells and Systems and MS Center Noord Nederland (MSCNN), University Medical Center Groningen, University Groningen, Groningen, The Netherlands; 12 Department Anatomy and Neuroscience, Amsterdam University Medical Center (V|UMC|), Amsterdam, Netherlands; 13 Department of Neurology and Immunology, Mayo College of Medicine and Science, Rochester, MN MN55905 USA; 14 Department of Neurosurgery, Freiburg University Medical Center, Freiburg, Germany; 15 Spinal Cord Injury Medicine, Department of Neurology, The Ohio State University, Wexner Medical Center, Columbus, USA; 16 Centre for Cardiovascular Science, Queens Medical Research Institute, 47 Little France Crescent, Edinburgh, UK; 17 Centre for Microsystems and Photonics, Electronic and Electrical Engineering, University of Strathclyde, Glasgow, UK; 18 Centre for Discovery Brain Science, Edinburgh Medical School, College of Medicine and Veterinary Medicine, University of Edinburgh, Edinburgh, UK; 19 Department of Neuroimmunology, Center for Brain Research, Medical University Vienna, Spitalgasse 4, 1090 Vienna, Austria

**Keywords:** Multiple sclerosis, Mitochondria, Demyelination and neuroprotection

## Abstract

Axonal loss is the key pathological substrate of neurological disability in demyelinating disorders, including multiple sclerosis (MS). However, the consequences of demyelination on neuronal and axonal biology are poorly understood. The abundance of mitochondria in demyelinated axons in MS raises the possibility that increased mitochondrial content serves as a compensatory response to demyelination. Here, we show that upon demyelination mitochondria move from the neuronal cell body to the demyelinated axon, increasing axonal mitochondrial content, which we term the axonal response of mitochondria to demyelination (ARMD). However, following demyelination axons degenerate before the homeostatic ARMD reaches its peak. Enhancement of ARMD, by targeting mitochondrial biogenesis and mitochondrial transport from the cell body to axon, protects acutely demyelinated axons from degeneration. To determine the relevance of ARMD to disease state, we examined MS autopsy tissue and found a positive correlation between mitochondrial content in demyelinated dorsal column axons and cytochrome *c* oxidase (complex IV) deficiency in dorsal root ganglia (DRG) neuronal cell bodies. We experimentally demyelinated DRG neuron-specific complex IV deficient mice, as established disease models do not recapitulate complex IV deficiency in neurons*,* and found that these mice are able to demonstrate ARMD, despite the mitochondrial perturbation*.* Enhancement of mitochondrial dynamics in complex IV deficient neurons protects the axon upon demyelination. Consequently, increased mobilisation of mitochondria from the neuronal cell body to the axon is a novel neuroprotective strategy for the vulnerable, acutely demyelinated axon. We propose that promoting ARMD is likely to be a crucial preceding step for implementing potential regenerative strategies for demyelinating disorders.

**Electronic supplementary material:**

The online version of this article (10.1007/s00401-020-02179-x) contains supplementary material, which is available to authorized users.

## Introduction

Demyelination leads to the damage and loss of axons and the progression of neurological disability in demyelinating disorders, including multiple sclerosis (MS) [17, 47, 70]. In inflammatory demyelinating disorders such as MS, axons are damaged during acute demyelination, and they can also degenerate over much longer timescale due to chronic lack of myelin [70]. There are no neuroprotective strategies to preserve the acutely demyelinated axon, highlighting a major and urgent unmet clinical need.

Myelination enables saltatory conduction of action potentials and conserves neuronal energy by clustering voltage-gated Na^+^ channels to the nodes of Ranvier [47]. In contrast, the demyelinated axon is bioenergetically challenged, and has an increased reliance on the Na^+^/K^+^-ATPase, and therefore ATP, to maintain the resting membrane potential and axonal integrity due to the redistribution of ion channels [76]. In particular, sodium channels redistribute along the axolemma following demyelination, which leads to an excess of sodium influx in to the axon. When ATP is not readily available, an excessively high sodium concentration in the axon leads to the reversal of Na^+^/Ca^2+^ exchanger and accumulation of calcium, which activates calcium dependent proteases and degeneration of the demyelinated axon [76]. One underlying mechanism that contributes to the degeneration of demyelinated axons, likely both acute and chronic, is an imbalance between the increased energy demand of nerve conduction and the generation of ATP in the demyelinated axon [37, 71].

ATP needed to meet the increased energy demand of the demyelinated axon is most efficiently produced by mitochondria. Neurons with healthy mitochondria respond to demyelination by increasing the mitochondrial content in acutely demyelinated axons [37]. The increased mitochondrial content of demyelinated axons is consistently evident in MS autopsy cases and experimental disease models [39, 45, 51, 57, 77, 81]. Increased axon damage in knockout mice, where the axonal mitochondrial content fails to increase following demyelination, due to the lack of a docking protein (syntaphilin), suggests that the neuropathological observation of increased mitochondrial content in demyelinated axons is an attempt to alleviate the energy imbalance [51].

The energy imbalance in demyelinating disorders is further exacerbated by the mitochondrial respiratory chain deficiency in neurons, which impairs the capacity to generate ATP [37]. In progressive MS, both clonally expanded mitochondrial DNA (mtDNA) deletions and decrease in nuclear DNA encoded mitochondrial transcripts in neuronal cell bodies have been implicated in the mitochondrial respiratory chain deficiency [6, 9, 14, 78]. The resulting mitochondrial deficiency is likely to hamper the energy producing capacity of demyelinated axons together with mitochondrial injury that is imposed by inflammation [49, 65]. It remains untested whether targeting of neuronal mitochondria with healthy and deficient respiratory chain function, might mitigate axonal loss following demyelination and address the key substrate of neurological disability—the vulnerable axon.

Against this background, we set out to study neuronal mitochondria in detail using cerebellar slices, microfluidic chambers, established experimental disease models as well as autopsy tissue and a novel in vivo model. We discovered a homeostatic response, which we termed the axonal response of mitochondria to demyelination (ARMD). Enhancement of ARMD therapeutically protected acutely demyelinated axons. Given the already recognised mitochondrial respiratory chain complex IV deficiency in cortical neurons in MS, we next studied dorsal root ganglia (DRG) neurons and their centrally projecting axons. We identified respiratory deficient DRG neurons and evidence of ARMD in disease. Upon modelling, we show that enhancing ARMD in respiratory deficient neurons, in vivo, protects these extremely vulnerable acutely demyelinated axons from degeneration.

## Materials and methods

### Preparation of cerebellar slice culture

Cerebellar slices were prepared as previously described [4]. Briefly, wild type C57BL/6 and *Shiverer* mice pups were sacrificed at P10 and cerebellum was placed in ice-cold dissection medium. The sagittal slices were then sectioned into 300 µm thick slices and placed on a membrane insert. Picospritzer III (Parker, US) and a micromanipulator were used to inject mEOS2-Lentivirus (titre 7–8 × 10^9^; aliquots stored at − 80 °C) containing 0.025% of Fast-Green (FG, Sigma F7258, UK) in to the Purkinje cell body layer. To enhance activation of the CMV-promoter driven mEOS2 construct, 10 µM of Forskolin (Forskolin *Coleus forskohlii*, 344282 Sigma) was added to the slice culture medium the day after injection and removed 2 days later. To demyelinate cerebellar slices, we used lysolecithin (l-*α*-lysophosphatidylcholine; L4129 Sigma), which minimally impacts mitochondrial function [4]. 0.75 mg/ml Lysolecithin was added to the medium for 17 h at DIV13. For the time course experiments of the ARMD, 0.5 mg/ml lysolecithin was added to the medium for 17 h. The slices were then fixed and stained using immunofluorescence histochemistry at several timepoints after removal of lysolecithin to determine changes in axonal mitochondrial parameters and axonal bulbs. Only demyelinated axons that were not transected were chosen for axonal mitochondrial analysis.

### Preparation of viral particles

Cloning and preparation of Lentivirus were performed by Pamela Brown as previously described [41]. Miro1 and PGC1α plasmids were sourced from Addgene (pRK5-Miro1, plasmid #47888; AAV-CMV-Flag-PGC1α-6His, plasmid #67637). Miro1 was inserted into a pDONR-P2A-mKate2 vector. The pDONR-P2A-mKate2 vector was used as a negative control and as an axonal marker in live imaging experiments. PGC1α was inserted into a pDONR-P2A-eGFP. The m1m4-eGFP (kind gift from Alan Peterson and Anna Williams) was amplified using primers with attB sites and cloned into a pDONR vector. All plasmids were shuttled into a lentiviral backbone pLenti6-cppt-delta CMV-DEST-opre, as described previously [11]. The viral titers were as follows mEOS2 8.2 × 10^9^ cfu/ml, Miro1 5 × 10^8^ cfu/ml, PGC1α 1.4 × 10^8^ cfu/ml, mKate2 2.25 × 10^9^ cfu/ml, m1m4-eGFP 6.74 × 10^9^ cfu/ml.

### Live imaging and analysis of axonal mitochondria in cerebellar slices and identification of demyelinated axons in-vitro using spectral confocal reflectance microscopy (SCoRe)

Live imaging of mitochondria in Purkinje cell axon was performed at DIV14, directly after removing the lysolecithin. Mitochondria in the most proximal 50 µm segment of the Purkinje cell axon were photoconverted using the 405 nm laser at 3% laser power for 20 s [34]. Immediately after photoconversion, the 85 µm long proximal axonal segment was imaged every minute for 20 min. An 8–12-μm stack was created from images every 0.5 μm in depth. Time lapse images were used to generate videos, exported from the Zeiss software, while all further analysis was done in Fiji [59]. Newly transported mitochondria, either appearing from the neuronal cell body to the most proximal 20 μm axonal segment or distal axonal mitochondria appearing in the 20 μm long axonal segment of the most distal photoconverted segment, were counted visually and confirmed using the kymograph. Green mitochondria represent newly transported mitochondria and red labelled mitochondria represent pre-existing mitochondria in the proximal axonal segment. For each mitochondrion appearing, the direction of movement was noted, as well as the area of the mitochondrion was measured manually, using the measure function in Fiji, when it first appeared in the axon.

Kymographs of the time lapse images were generated by using the ImageJ plugin KymographClear2.0 [40]. Mitochondrial speed of movement was determined by using the Kymotoolbox ImageJ plugin [80]. The mitochondria moving anterograde from the cell body to the axon were identified by the slope direction of tracks within the 20 microm of the kymograph and subsequently confirmed on the video. For retrograde moving mitochondria 20 microm of the most distal part was used.

We used spectral confocal reflectance microscopy (SCoRe) to determine the myelination status of the axons [58]. Specificity of SCoRe, to differentiate demyelinated axons from myelinated axons, was confirmed by immunofluorescence staining for Myelin Basic Protein (MBP) and neurofilament and then overlapping it with SCoRe image.

### Triple immunfluorescent staining and confocal imaging of cerebellar slices

The membrane inserts containing cerebellar slices were cut out and fixed in PFA before heat mediated antigen retrieval. After blocking with Normal goat serum (NGS, Vector S-1000, US), the free floating slices were incubated with three primary antibodies (Supplementary Table 1, online resource), and then exposed to secondary antibodies, before mounting using Vectashield with DAPI (Vector H-1200, US) [52]. Confocal images of triple staining (Supplementary Table 1, online resource) were acquired on a Zeiss LSM 710 inverted confocal microscope (Zeiss, Germany).

To quantitate axonal mitochondria, images were processed in Adobe Photoshop CS6 (Adobe, US) and all non-axonal mitochondria (outside the NF + structures) were removed. Images were then opened in Fiji and individual axons were cut out to analyse the axonal mitochondrial parameters using macros. The first macro split the images into the separate color channels, the second macro measured the axonal area and the third macro measured the axonal mitochondrial number and area. For each slice, 40 axons were chosen and the mean axonal mitochondrial occupancy was calculated for each data point, which represents a slice from a different animal.

For the analysis of axonal bulbs, images of NF labelling were acquired as mentioned above and the axonal bulbs per field of view in × 63 images were counted. For each cerebellar slice five fields of view were randomly selected and the axonal bulb were counted and averaged for each datapoint.

### Triple immunofluorescent staining and confocal imaging of mouse spinal cord and MS cryosections

Longitudinal cryosections, 15 microm thickness, of dorsal spinal cord were placed on glass slides and stored at − 80 °C. The triple immunofluorescent staining and confocal images were taken from wild type and mutant mice as well as human tissue and followed the same protocol as for the cerebellar slices.

For the analysis of axonal mitochondria, only the non-transected dorsal column axons were included. Acutely demyelinated experimental lesions were identified by DAPI staining and loss of MBP staining. Chronic MS lesions were identified by loss of MBP and serial sections were used for the mitochondrial analysis. Images were processed and analysed as described for cerebellar slices. For calculating mitochondrial complex IV deficiency in mouse spinal cord axons (wild type and mutant) and human dorsal column axons, the mitochondrial channels of complex II 70 kDa and COX-I were merged in Fiji. A macro was used to calculate the percentage of complex II 70KDa-positive regions that were co-labelled by COX-I for each axon. For each case, 40 axons were chosen and the mean axonal mitochondrial occupancy was calculated for each data point, which represents a different animal or human case. Axonal bulbs were quantitated as described for cerebellar slices.

For the analysis of axon number in dorsal columns of WT and COX10Adv mutant mice, frozen transverse sections of cervical spinal cord were fixed and stained, as described previously. The sections were then imaged on a Zeiss ApoTome.2 (Zeiss, Germany) with tile scan function and stitching to generate a single image file for the whole dorsal column. The total axon number per animal was determined using “analyse particle” function in FIJI. For the analysis of PGC1α-positive nuclei in DRG neurons, snap frozen spinal cord was cryosectioned longitudinally, fixed and stained for PGC1α, NF200 and peripherin. Images of the whole DRG were acquired and the percentage of PGC1α + NF200 + cells was determined. For the analysis of mitochondrial respiratory chain subunits of the mitochondrial respiratory chain within human DRG neurons, we triple stained using NF200, complex II 70 kDa and a number of subunits (Supplementary Table 1, online resource). The percentage of DRG neurons that were deficient in mitochondrial respiratory chain subunits was determined in serial sections of human DRG.

### Design and fabrication of microfluidic chambers

Microfluidic chambers were fabricated in polydimethylsiloxane (PDMS) (Sylgard 184, Dow Corning, US) using standard soft lithography techniques, comprising of an array of microchannels between two culture chambers that are fluidically addressable via inlet/outlet wells, as previously described [36]. Prior to use, these devices were coated with 0.45 mg/ml Matrigel (Corning 356231, US) for 1 h at RT and 30 mg/ml Poly-D-lysine (PDL, Sigma Aldrich P6407, UK) for 30 min at RT. To achieve fluidic isolation in a given chamber we added at least 10 µl more of the medium to the opposite chamber, which prevents diffusion from the treated to the untreated chamber, as previously shown [55, 56].

### Culture of DRG neurons and OPCs in microfluidic chambers

DRG neurons were rapidly extracted from P4-P8 old C57BL/6 mice pups, as previously described [64]. Differential adhesion was used to remove excess glial cells from the culture. After seeding the DRGs in seeding medium a concentration of 20 µM FUDR was added to the DRG neuronal cell body chamber and the axonal chamber to reduce growth of non-neuronal cells. A concentration gradient of 25 ng/ml NGF in the cell body chamber to 50 ng/ml NGF in axonal side was created to enhance axonal growth. The day after placing DRG neurons in the cell body chamber, the seeding medium was completely removed and replaced with maintenance medium. Until the 10 day after seeding, FUDR was added at a concentration of 10 µM to both sides of the chamber, while the NGF gradient was maintained for the same period. At 10 days after seeding Lentivirus expressing mKate2 was added to the cell body chamber at MOI 25 to label DRG neurons in the cell body chamber and their axons in the axonal chamber.

OPCs for myelinating cultures were obtained by dissection of Sprague Dawley rat cortices, as previously described [82]. Once cell count was done using a hemocytometer, M1-M4-eGFP lentivirus was added at MOI 40 to approximately 100,000 OPC. Oligodendrocyte precursor cells (OPCs) were seeded into the axonal chamber at 12 days following DRG seeding in the cell body chamber, in order to allow sufficient number of axons to have crossed into the cell body chamber [72]. Immediately following seeding of OPCs, the maintenance medium in both chambers was replaced with myelination medium [72]. Thereafter co-cultures were maintained for another 2 weeks, while renewing the myelination medium two times a week, to ensure adequate myelination prior to the visualization of myelinated axonal segments in live-imaging.

### Demyelination of microfluidic chambers and analysis of axonal damage and myelin-status in live images

We established that 0.005 mg/ml of lysolecithin for 2 h was sufficient to demyelinate DRG-OPC co-cultures without damaging the DRG axons in unmyelinated cultures. Before adding lysolecithin to microfluidic chambers, we used an Axio Observer Z1 inverted motorized microscope (Zeiss, Germany), for live imaging to identify the myelinated axonal segments (video 11, online resource). Images of the myelinated axonal segments were saved with *x*–*y* co-ordinates of the stage position. The effect of live imaging on the axons and myelin was determined by imaging microfluidic chamber containing both mKate2 expressing DRGs and eGFP-expressing OPC where imaged at 20 × magnification (Plan-Apochromat 0.8 NA Ph 2 M27 objective, Zeiss, Germany) for 30 min before returning the microfluidic chambers to the incubator. Re-imaging 24 h later showed that there was no significant effect of live imaging on axonal health.

We then added lysolecithin for 2 h before live imaging the previously imaged axonal segments again, using the *x*–*y* co-ordinate of the stage position. These live images of axons, pre and post lysolecithin, allow us to assess the demyelination of the axonal segments (based on M1-M4-eGFP fluorescence) as well as axonal damage following acute demyelination (based on appearance of mKate2 fluorescence). Axonal structure was categorized as intact, beaded or fragmented, both prior to and following exposure to lysolecithin. Intact axons showed continuous mKate2 fluorescence and beaded axons showed obvious irregularities in axon diameter without transection. Meanwhile, axons were classed as fragmented when the mKate2 fluorescence was disrupted and not continuous in at least one part of the axon. Nearly all myelinated axonal segments were intact prior to exposure to lysolecithin and only intact myelinated axons were considered for the assessment of axon damage following acute demyelination. Following exposure to lysolecithin, demyelination was confirmed based on disruption or loss of M1-M4-eGFP fluorescence. On average 12 myelinated axonal segments were included per microfluidic chamber. An average of data from 2–3 microfluidic chambers per batch of experiments were pooled to generate a single data point presented in Fig. 3.

### Manipulating mitochondrial dynamics in unmyelinated and myelinated cultures

We targeted anterograde movement of mitochondria in unmyelinated DRG neurons, by over expression Mitochondrial Rho GTPase1 (Miro1), using a lentivirus [22]. Furthermore, we targeted mitochondrial biogenesis in neurons by over expressing peroxisome proliferator-activated receptor gamma (PPAR-γ) coactivator 1-alpha (PGC1α), using a lentivirus [25, 54]. We then pharmacologically targeted mitochondrial biogenesis in neurons by using pioglitazone [44]. DRG were extracted as described previously and seeded on glass-bottomed 35 mm dish (µ-dish35mm, low Grid-500 ibiTreat, ibidi 80,156, Germany). Lentivirus Miro1 at MOI 10 was added to the culture medium at seeding (3 weeks before live imaging) and Lentivirus PGC1α at MOI 10 was added at 2 and 4 days and 3 weeks before live imaging. Finally, 2 µM pioglitazone was added at 2, 4 and 6 days and 3 weeks before live imaging and renewed with each media change. The same manipulations were carried out in DRG neurons co-cultured with OPCs, which were added DIV12. Myelinated axonal segments were identified for confocal imaging using SCoRe, as previously described for cerebellar slices (Supplementary Fig. 1, online resource).

### Manipulating axonal mitochondria by targeting DRG neuronal cell bodies in microfluidic chambers

In order to avoid pioglitazone impacting oligodendrocyte lineage cells and myelinated axons, we applied the drug to the DRG neuronal cell body chamber at a concentration of 2 µM for 6 days prior to demyelination. Pioglitazone was renewed in the neuronal cell body chamber with each media change. Furthermore, PGC1α inhibitor [15 µM SR-18292 (SML2146, Sigma UK)] was added together with pioglitazone to neuronal cell body chamber [62]. Following 6 days of pioglitazone treatment of the neuronal cell bodies, with or without PGC1α inhibitor.

Following 6 days of pioglitazone treatment of the neuronal cell bodies we added lysolecithin to the axonal chamber to demyelinate, as previously described.

Lentivirus Miro1 and Lentivirus PGC1α were applied to the neuronal cell body chamber at seeding and DIV16, respectively. At DIV26, lysolecithin was added to the myelinating chamber, as previously described. Axonal integrity of myelinated segments (before demyelination) and axonal damage following demyelination (of the same myelinated axonal segment) were quantified using live imaging, as previously described.

### Manipulating axonal mitochondria in cerebellar slices

The cerebellar slices from wild type C57BL/6 mice pups (P10) were prepared as described and the slices were maintained on membrane inserts in culture for a week, before adding 40 µM pioglitazone (Sigma PHR1632, UK) to the culture medium. Two days following exposure to pioglitazone, culture medium was renewed and lysolecithin 0.5 mg/ml was added to the culture medium for 17 h. Following the removal of lysolecithin, pioglitazone was replaced in the culture medium for 3 days until fixing and staining of the slices, as previously described. Triple immunofluorescent labelling and confocal microscopy were used to assess demyelination and axonal bulb formation as well as mitochondrial occupancy of non-transected and demyelinated axons, as previously described.

### Photoconversion of mEOS2 and live imaging of axonal mitochondria in DRG neurons

Live imaging of mitochondria located in the proximal segment of DRG axon, was performed with or without mitochondrial manipulations, as described above for cerebellar slices. Each data point on the graphs in Fig. 2 represents the value of a single axon.

To assess the ARMD response, DRG neurons were seeded in microfluidic chambers and mEOS2 lentivirus was added at seeding. At DIV12, OPC were added to the axonal chamber to achieve myelination, as described previously. At DIV24, the chambers were imaged on a Leica SP8 microscope with temperature control at 37 °C and 5% CO_2_ flow with a 25 × water immersion objective (Leica). Per chamber all mEOS2 positive mitochondria in axons within two fields of view in the axonal chamber and the adjacent microchannels were then converted using the 405 nm laser at 3% laserpower for 2 min. To assess ARMD chambers were demyelinated using 0.005 mg/ml lysolecithin for 2 h. The chambers were then returned to the incubator overnight. The following day photoconverted regions were imaged to assess newly transported mitochondria (green) from the cell body chamber to the axonal chamber. SCoRe was used to determine the myelinated status of the axons. To analyse the amount of newly transported green mitochondria in the axonal chamber, 20 axons were randomly selected, cut out and saved as single axon images. Fiji was then used to calculate the proportion of green in red area. To calculate the axonal mitochondrial occupancy, the “analyse particles” function of Fiji was used to determine the mitochondrial area, which was then corrected for the length of the axonal segment. Each dot in Supplementary Fig. 3, online resource, represents an axon.

### Assessing the effect of mitochondrial manipulations on lysosomal trafficking in unmyelinated DRG neurons

To determine the effect of Miro1, PGC1α and pioglitazone on lysosomal trafficking in DRG neurons, unmyelinated DRG neurons were seeded on glass-bottomed 35 mm dishes, as previously described. Miro1 was added at seeding, while PGC1α and pioglitazone were added to the DRG neurons at 10 days and 6 days before imaging, respectively. At DIV14 LysoTracker (LysoTracker Red DND-99, L7528 Invitrogen) was added to the culture medium at a concentration of 50 nM and incubated for 30 min at 37 °C, before washing the LysoTracker off and adding live imaging solution to the DRG neurons. Live fluorescence imaging was performed as described for microfluidic chambers, using a 63 × oil immersion objective (Plan-Apochromat 1.40 NA Oil DIC M27 objective, Zeiss, Germany). Videos and Kymographs were generated as described previously. The total number of lysosomes moving in both directions were counted on the kymograph and visually confirmed on the videos and the direction of movement for every single lysosome was noted. Each datapoint on Fig. 2f and g represents the average number of moving lysosomes per axon.

### Pharmacological inhibition of complex IV in DRG neurons using sodium azide

Sodium azide (Sigma S8032, UK) was used to inhibit complex IV in unmyelinated DRG neurons, cultured on glass bottom dishes, as described previously [39]. Firstly, a concentration gradient experiment was performed to determine the sublethal sodium azide (Sigma S8032, UK) dose for DRG neurons, which inhibits complex IV, by trypan blue exclusion test [67]. The highest dose, which did not impact cell viability and resulted in complex IV inhibition, was determined as 100 µM sodium azide for 17 h, which was used for all the subsequent complex IV inhibition experiments. Complex IV histochemistry was performed as described below and images were obtained using bright field microscopy. The intensity of the complex IV reactive product was assessed using FIJI and densitometry to analyse complex IV activity at a single cell level.

For live imaging of mitochondrial dynamics in complex IV deficient DRG neurons, we cultured unmyelinated DRG neurons on glass bottom dishes, as described previously. Lentivirus mEOS2 was added at seeding and live imaging was performed at DIV21, as described previously. Kymograph were prepared and mitochondria were analysis as described previously for Purkinje cell axons in cerebellar slices.

### Complex IV inhibition and Seahorse analysis of mitochondrial respiration

DRG neuronal cells were cultured on V7 Seahorse 24-well cell culture microplates (Agilent Technologies), in maintenance medium in a 5% CO_2_ 37 °C incubator. Sodium azide was present in media at either 0.1 or 1 mM for 17 h before the Seahorse experiment, and the sodium azide was maintained in the media during the Seahorse run. Plates were incubated for 30 min at 37 °C (without CO_2_), before entry into the Seahorse XFe24 Extracellular Flux Analyser (Agilent). Three measurements were taken basally, and three measurements taken after injection of each drug to either inhibit ATP-linked respiration, uncouple respiration or inhibit the respiratory chain. Mitochondrial respiration was calculated by subtracting the first OCR measurement following injection of antimycin/rotenone from the third basal OCR measurement. Normalisation of OCR to relative protein content was achieved following Sulforhodamine B (SRB) staining of all cell wells. Data for each treatment groups was averaged from between 4 and 5 replicate wells.

### Human tissue

We obtained frozen human autopsy tissue, including dorsal root ganglia and spinal cord blocks, from the rapid autopsy program at Cleveland Clinic, Ohio, USA and Netherland Brain Bank (Table 1). The frozen tissue blocks were stored at − 80 °C until cryosectioning. The entire DRG were cryosectioned at 15 microm intervals. Cryosections were then subjected to COX/SDH histochemistry, COX/immunofluorescent labeling as well as laser micro dissection of single neurons, as described below.

**Table 1:** Details of human autopsy cases

Case no	Age/gender	Subtype	Disease duration	Cause of death	PMD	C and L	
MS1	34/M	MS			< 24	C3	L3
MS2	41/F	SPMS	11	End stage MS (natural death)	8	C6	L2
MS3	56/M	SPMS	14	GI bleed	10	C6	
MS4	64/M	PPMS	34	End stage MS (natural death)	8		L1
MS5	69/F	SPMS	26	Viral infection	13	C5	L3
MS6	77/F	Progressive MS	> 6 years	Pneumonia	7		L2
MS7	40/F	Progressive relapsing MS	8	Haematemesis	9		L5
MS8	50/F	SPMS	17	Euthanasia	7	C8	L1
MS9	53/M	SPMS	9	Cardiopulmonary failure	5		L5
MS 10	61/M	SPMS	31	Euthanasia	9		L5
MS 11	71/F	Progressive MS	23	Respiratory failure	10		L2
MS 12	78/F	SPMS	25	Cerebrovascular accident	11	C7	L3
MS 13	81/F	SPMS	59	Pneumonia	7		L1
MS 14	82/M	SPMS	32	Pneumonia	6	C8	
MS 15	88/F	PPMS	25	Chronic colitis	8		L5
MS 16	62/F	SPMS	43	Pneumonia	6		L
MS 17	70/M	SPMS	46	Cardiac arrest	7		L2
MS 18	74/M	SPMS	36	Respiratory failure	9		L
CON 1	74/M	–	–	Lung carcinoma	7		L2
CON 2	58/M	–	–	Oligodendroglioma (left parieto-occipital)	5	C	L2
CON 3	71/F	–	–	Renal failure	7	C	L
CON 4	68/F	–	–	Sepsis	42		L
CON 5	70/M	–	–	Cerebrovascular accident	24		L
CON 6	55/F	–	–	Sepsis	24		L
CON 7	62/F	–	–	Euthanasia (renal cell carcinoma)	8		L2
CON 8	80/M	–	–	Cardiac failure	8	C	L1
CON 9	92/F	–	–	Urosepsis	7		L1
CON 10	84/M	–	–	Cardiopulmonary failure	5		L2
CON 11	71/M	–	–	Pneumonia	7	C	
CON 12	47/F	–	–	Breast carcinoma	4	C	
PD 1	84/M	–	–	Pneumonia	7		L
PD 2	84/F	–	–	Old age	6		L
PD 3	79/F	–	–	Renal failure	5		L
MND 1	58/M	–	–	Respiratory failure	6		L
MND 2	74/M	–	–	Pneumonia	7		L

*C* cervical, *CON* control, *L* lumbar, *MND* motor neuron disease, *MS* multiple sclerosis, *PD* Parkinson’s disease, *PMD* post mortem delay, *SPMS* secondary progressive MS. Human autopsy tissue was obtained from Cleveland Clinic, Netherland Brain Bank and Edinburgh Brain Bank

### Complex IV/complex II histochemistry and analysis of complex IV deficient DRG neurons

Mitochondrial respiratory chain complex IV(COX)/complex II (succinate dehydrogenase [SDH]) activity was assessed using the well-established sequential COX/SDH histochemistry, as previously described [9]. In five randomly chosen DRG cryosections, complex IV deficient neurons (stained blue) were calculated as a percentage of total neurons (sum of neurons stained either brown or blue) in both frozen human (Table 1) and frozen mouse tissue (Table 2 and COX10Adv mutant mice). For the detection of complex IV deficient cells with intact complex II within the brain spinal cord of the animal models (Table 2), we stained every 5th section of the entire brain and spinal cord and scanned the sections at 40 × objective manually to look for cells stained blue (complex IV deficient with intact complex II).

**Table 2:** Details of established experimental disease models

Model (co-author)	Species (strain)	Time points for analysis in days	*n* = (brain, spinal cord, DRG)
Focal demyelinating dorsal funiculus lesion: LPC (1%) [79]	Mouse (C57BL/6)	5*	0, 6, 6
LPS (200 ng) [16]	Rat (Sprague–Dawley)	7*	0, 6, 3
Cuprizone-mediated demyelination of the brain [21]	Mouse (C57BL/6)	42* 91	6, 0, 0 6, 0, 0
TMEV-induced inflammatory demyelination [57]	Mouse (SJL/J)	7 (acute encephalitis) 41 (demyelinating) 112 (axonal loss)* 209 (chronic)	3, 3, 3 3, 3, 3 3, 3, 3 3, 3, 3
T-reg depleted active EAE with MOG_35–55_ [42]	Mouse (C57BL/6)	13 (acute)* 30 (resolution) 60 (chronic)	0,10, 0 0, 7, 0 0,9, 0
Humanized TCR transgenic with spontaneous EAE [15]	Mouse (C57BL/6)	120–150*, (clinical score 1–2 and > 3)	3, 3, 0 3, 3, 0
Chronic EAE with subcutaneous spinal cord homogenate [2]	Mouse (Biozzi ABH)	18 (acute)* 35–40 (relapsing) 120 (chronic)	3, 3, 3 3, 3, 3 10, 10, 6
Acute EAE with rMOG [12]	Rat (Dark Agouti, Harlan)	1 3 14*	3, 3, 0 3, 3, 0 3, 3, 3
EAE with rMOG_34–56_ [26]	Marmoset (*Callithrix jacchus)*	11 days*, on average, post EAE score of 2.5	9, 5, 2

*DRG* dorsal root ganglia. *EAE* experimental autoimmune encephalomyelitis, *LPC* lysolecithin, *LPS* lipopolysaccharide, *MOG* myelin oligodendrocyte glycoprotein, *TCR* T-cell receptor, *TMEV* Theiler’s murine encephalomyelitis virus, *n* number of animals used for brain and spinal cord analysis

*Indicates peak clinical disease or peak demyelination time point for the analysis of axonal mitochondrial parameters. All the time points stated above were included in the detection for respiratory-deficient cells. Equal numbers of age-matched controls were used, except for marmoset EAE where 4 age-matched controls were used

### Sequential COX/immunofluorescent labeling of DRG neurons and CNS cells

To identify complex IV in proprioceptive and nociceptive DRG neurons, NF200 and peripherin were immunofluorescently labeled following completion of complex IV histochemistry step, as previously described [38]. Both brightfield and fluorescent images of five randomly chosen DRG cryosections were obtained using Zeiss ApoTome.2 microscope (Zeiss, Germany) and superimposed using ImageJ to identify complex IV activity within proprioceptive and nociceptive DRG neurons. Proprioceptive neurons were identified as NF200 + peripherin-. To identify complex IV deficient CNS cells in animal models, independent of complex II activity, we performed sequential COX/immunofluorescent histochemistry and used antibodies against COX-I and complex II 70 kDa (Supplementary Table 1, online resource), as previously described [38]. To look for immunofluorescently labelled cells, we stained every 5th section of the entire brain and spinal cord, except marmoset EAE, where tissue blocks were used, and manually scanned the sections at 40 × objective.

### Laser capture of single DRG neurons from MS tissue and grey matter from animal models for Real time PCR and long range PCR

Cryosections (15 μm thick) were mounted onto membrane slides (Leica) for laser micro dissection. Following COX/SDH histochemistry, single DRG neurons were micro dissected using a Leica laser micro dissection microscope (Leica LMD), as previously described [9]. Spinal cord grey matter regions from snap frozen tissue was micro dissected from animal models (Table 2), except in cuprizone model where approximately 250 × 250 μm^2^ region of cortex was included. DNA extraction was carried out using the QIAamp DNA Micro Kit (Qiagen).

To analyse the level of mitochondrial DNA deletion in single DRG neurons in MS tissue we used a real-time PCR, as previously described [9]. Known deletion-level standards, a blood-positive control and a blood-negative control, run in triplicate, were added to the assays. To detect mtDNA deletions in human autopsy tissue and in snap frozen tissue from animal models, we used long-range PCR, as previously described [9].

### Cresyl violet staining of DRG neurons

One in every five serial sections of each DRG was processed using cresyl violet staining and the total number of DRG neurons with nuclei was counted per DRG section for each animal and human case. An average of 4–5 DRG per animal and human case was included in each data point shown in Supplementary Fig. 6a–c, online resource and Supplementary Fig. 10a, online resource.

### Animal genotyping and focal lesioning of the spinal cord dorsal columns

To generate inducible knockout of complex IV subunit 10 (COX10) in DRG neurons, we crossed COX10^flox/flox^ mice with Advillin^CreERT2/+^ mice to derive COX10^flox/flox^Advillin^CreERT2/+^ mutant mice (COX10Adv mutants) [13, 48]. Wild type and COX10Adv mutant mice were maintained on C57Bl6 background. Mice were genotyped, as previously described [13, 48]. Tamoxifen was dissolved in sunflower oil/ at 20 mg/ml and gavaged at a dose of 60ul/10 g body weight daily over 5 consecutive days. On an Applied Biosystems 7500 Fast Real Time PCR system in triplicates, qPCR was performed using 10 ng of genomic DNA in a 12.5-μl assay using PowerSYBR Green PCR Master Mix (Applied Biosystems). A 167-base-pair *Cox10*^*flox*^-specific fragment was obtained with primers 5′-CGGGGATCAATTCGAGCTCGCC-3′ and 5′-CACTGACGCAGCGCCAGCATCTT-3′. All animal experiments were performed in compliance with Animals (scientific procedures) Act 1986 and UK Home Office guidelines under the animal license (PPL 70/7872). Tissue from established animal models was obtained through collaborations as listed in Table 2.

**Fig. 1:**
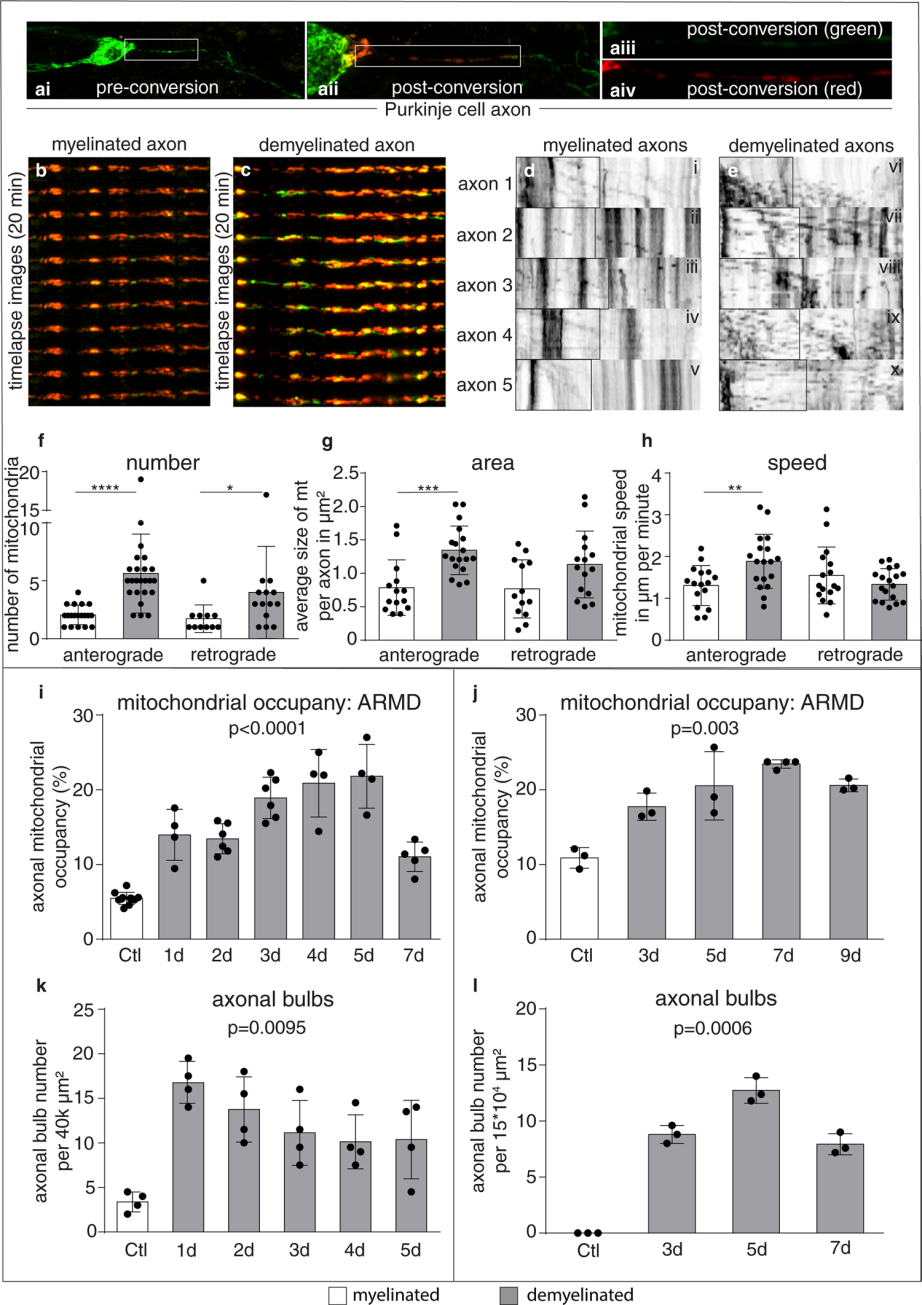
Demyelination mobilises mitochondria from the neuronal cell body to the axon and gradually increases the axonal mitochondrial content. **a** Mitochondria within Purkinje cells are labelled in live cerebellar slice cultures, using lentivirus-mitochondria-targeted mEOS2 (**a**i, green). Photoconversion of mitochondria within the proximal axon segment adjacent to the Purkinje cell body [green to red conversion in the region of interest (ROI) shown in **a**ii–iv], enables the tracking of mitochondria from the cell body to the axon (anterograde, left to right). **b-c** Following photoconversion, time lapse imaging of the ROI over 20 min shows newly transported mitochondria (green) amongst the photoconverted mitochondria (red) in a myelinated axon (**b**) and a demyelinated axon (**c**). SCoRe was used to determine the myelination status of the mEOS2 labeled axons (Supplementary Fig. 1, online resource). The majority of the photoconverted mitochondria remained stationary (**b, c**, red), while the newly transported mitochondria to the axon (**b, c**, green) continued to move (see videos for confirmation, online resource) and sometimes co-localised with the stationary mitochondria (presumably fused). **d, e** Kymographs of the green fluorescence channel show an abundance of mitochondria moving from the cell body to the ROI (left to right, anterograde transport) in demyelinated axons (**e**) compared with myelinated axons (**d**). See videos 1–5, online resource, for myelinated axons and videos 6–10, online resource, for demyelinated axons. **f–h** Quantitation of newly transported mitochondria (green) from the cell body to the ROI shows a significant increase in the number (**f**), area (**g**) and speed (**h**) of motile mitochondria in the demyelinated axons compared with myelinated axons. The number of retrograde moving mitochondria from the distal demyelinated axon segment to the ROI is also significantly increased upon demyelination (**f**). Data presented as dot-plot with mean (bar) ± standard deviation (whiskers). *p < 0.05, **p < 0.01, ***p < 0.001, ****p < 0.0001 using Mann–Whitney *U* test. **i, j** Mitochondrial content of acutely demyelinated and non-transected axons gradually increases and peaks at 5 days in cerebellar slice cultures, following exposure to lysolecithin for 17 h (**i**). The same peak is observed at 7 days, in vivo, following focal lysolecithin injection to the spinal cord (dorsal columns) of wild type mice (**j**). Each data point indicates the mean value of 20 axons from slice preparations or each animal. Statistical significance was determined using Kruskal–Wallis test. **k, l** Axonal injury following demyelination, judged by axon bulbs (transected axons), peaks 2–4 days earlier than the mitochondrial content in demyelinated axons in both cerebellar slices (**k**) and in vivo (**l**). Statistical significance was determined using Kruskal–Wallis test. ARMD: axonal mitochondrial response to demyelination

Both wild type mice and COX10Adv mutant mice of C57Bl6 background, aged approximately 13 weeks, were anaesthetized using inhalation of 3–4% isoflurane/oxygen with supplementation of 0.05 ml of buprenorphine administered subcutaneously. Following exposure of spinal vertebrae at T12/T13, a dorsal laminectomy was performed to expose the dura and the central vein. Dura just lateral to the central vein was pierced using a sterile dental needle. The tip of a pulled glass capillary, attached to a Hamilton syringe, was introduced into the dorsal column through the pieced dura at an angle of 45 degree, approximately, and 0.05 μl of 1% lysolecithin was injected to cause focal demyelination of the dorsal column in mice. Wild type mice were euthanased at 3, 5, 7 and 9 days post lesioning for the time course experiments (Fig. 1) and both wild type mice and COX10Adv mice were euthanased at 3 days post lesioning for the axon protection experiments (Figs. 3 and 6).

**Fig. 2:**
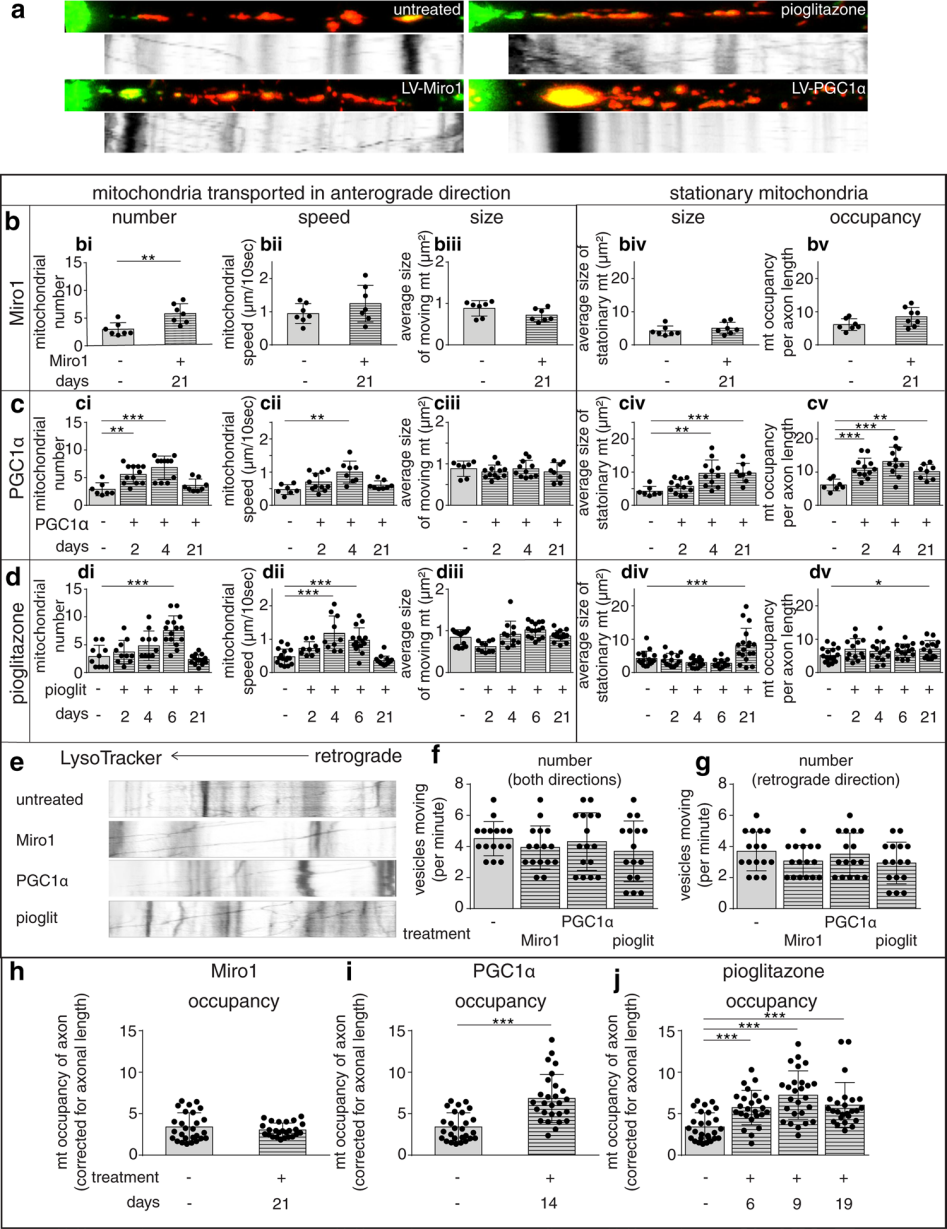
Enhanced mobilisation of mitochondria from the neuronal cell body to the axon by over-expression of Miro1 and targeting PGC1α/PPAR-γ pathway. **a** Following photoconversion of the mEOS2 labeled mitochondria in the proximal axon segment (green–red), time lapse images indicate the anterograde movement of newly transported mitochondria from the unmyelinated DRG neuronal cell body to the proximal axon segment (left to right, see videos, online resource). Over-expression of Mitochondrial Rho GTPase 1 (Miro1) and peroxisome proliferator-activated receptor gamma coactivator 1-alpha (PGC1α) in DRG neurons in culture using lentiviruses as well as exposure of DRG neurons to 2 µM pioglitazone, a thiazolidinedione, enhances anterograde transport of mitochondria, as shown in kymographs and videos, online resource. **b–d** Quantitation of the newly transported mitochondria in the proximal axon segment of unmyelinated axons (green) indicates a significant increase in the number of mitochondria mobilising from the DRG neuronal cell body to the proximal axon segment following over-expression of Miro1 (**b**) and PGC1α (**c**) and exposure to pioglitazone (**d**) compared with untreated DRG neurons (ctl: control). The speed of anterograde moving mitochondria is significantly greater with the over-expression of PGC1α and exposure to pioglitazone. The size of the anterograde moving mitochondria remained unchanged. Unlike Miro1, both PGC1α over-expression in DRG neurons and exposure to 2 µM pioglitazone significantly increase the size of the stationary mitochondria (red), and the total axonal mitochondrial content. **e–g** PGC1α and Miro1 over-expression as well as the application of pioglitazone to DRG neurons did not significantly alter the anterograde and retrograde transport of lysosomes within unmyelinated axons. **h-j** Similar to unmyelinated axons, Miro1 over-expression does not significantly alter the mitochondrial content within myelinated axons, whilst both PGC1α over-expression and application of pioglitazone significantly increase the axonal mitochondrial content within myelinated axons, in vitro. Data presented as dot-plot with mean (bar) ± standard deviation (whiskers). *p < 0.05, **p < 0.01 and ***p < 0.001 using Mann–Whitney *U* test once Kruskal–Wallis test showed a *p* < 0.05 in multiple subgroup comparisons

**Fig. 3:**
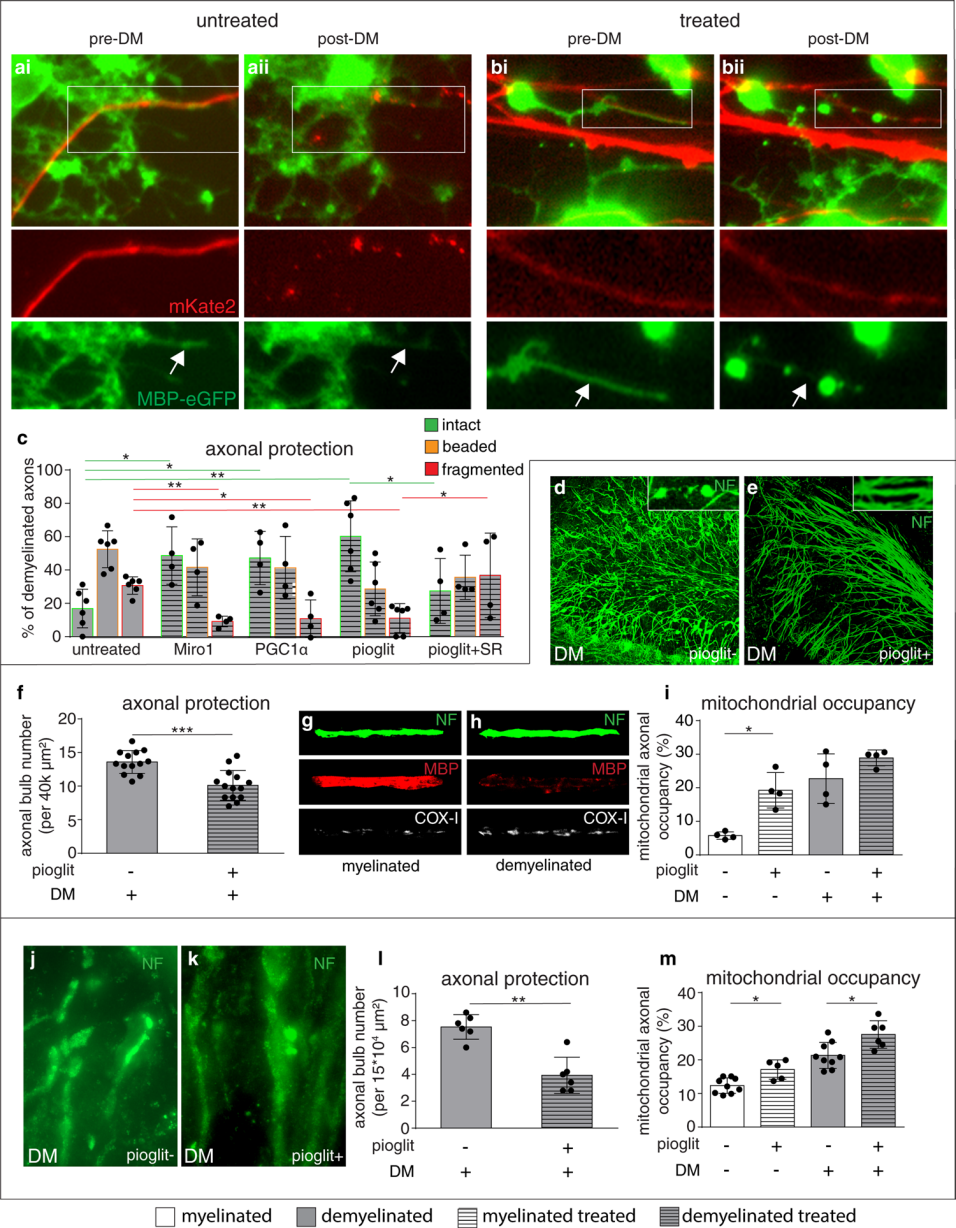
Enhancement of ARMD in wild type neurons, in vitro and in vivo*,* protects the acutely demyelinated axons. **a, b** We labelled dorsal root ganglia (DRG) neurons by applying lentivirus-mKate2 (red) to the cell body chamber while MBP produced by oligodendrocytes in the other chamber were labeled using lentivirus M1-M4 (green) (**a, b**). Prior to demyelination (pre-DM), live images identify myelinated axonal segments in the co-culture chamber (**a**i and **b**i, arrows). Following demyelination (post-DM), by exposing to lysolecithin for 2 h, live imaging shows damaged MBP-positive structures (**a**ii and **b**ii, arrows). We then targeted mitochondria in neurons by applying lentivirus-Miro1, lentivirus-PGC1α and pioglitazone to the neuronal cell body chamber (treated, shown in **b**i and **b**ii), prior to demyelination. All three manipulations protected the acutely demyelinated axonal segments (**b**, red) compared with untreated co-culture chambers (**a**, red). **c** For quantitation, axons were identified as intact (green outlined bar charts), beaded (orange outlined bar charts) and fragmented (red outlined bar charts) based on mKate2 signal, both prior to and following demyelination. Quantitation of axon injury following demyelination revealed a significant decrease in the proportion of demyelinated axons that are fragmented and a significant increase in demyelinated axons that remain intact, when the neuronal cell bodies were exposed to lentivirus-Miro1, lentiviris-PGC1α and pioglitazone (**c**). PGC1α inhibitor significantly diminished the protective effect of pioglitazone on demyelinated axons. Controls shown were exposed to lentivirus-mKate2. *p < 0.05 and **p < 0.01 using Mann–Whitney *U* test, once Kruskal–Wallis test showed a *p* < 0.05 in multiple subgroup comparisons.d**D–i** We detected axon bulbs (**d**, insert) when cerebellar slice cultures were demyelinated using lysolecithin (0.5 mg/ml) for 17 h (**d**). Application of 40 μM pioglitazone to cerebellar slice cultures prior to lysolecithin-induced demyelination significantly decreased the extent of axon bulb formation compared with solvent (DMSO) only controls (**f**). Axonal mitochondrial content increased upon demyelination of cerebellar slices (**g**, **h**), consistent with homeostatic ARMD. The application of pioglitazone significantly increased the mitochondrial content of myelinated axons (**i**, pioglit + lyso-), compared with untreated cerebellar slices (**i**, pioglit-lyso-). ***p < 0.001 using Mann–Whitney *U* test. **j–m** Demyelination of the spinal cord of wild type mice in vivo, using focal injection of lysolecithin (DM pioglit-) to the dorsal column, increases axon bulb formation (**j**) compared with non-demyelinated wild type mice (not shown) at 3 days post lesioning. Pioglitazone in diet for 6 weeks (120 mg/kg diet), prior to focal spinal cord demyelination, significantly decreased axon bulb formation in wild type mice (**k**, DM pioglit + and **l**) compared with controls on chow diet (**j**, DM pioglit- and **l**). We did not detect a significant change in DAPI + cells and Iba1 + cells in lesions with pioglitazone treatment (Supplementary Fig. 14, online resource). Mitochondrial content of both myelinated (**m**, DM-) and demyelinated axons (**m**, DM +) significantly increased following dietary pioglitazone in wild type mice (**m**, pioglit +) compared with control (**m**, pioglit-). Data presented as dot-plot with mean (bar) ± standard deviation (whiskers). *p < 0.05 and **p < 0.01 using Mann–Whitney *U* test. *DM* demyelinated, *MBP* myelin basic protein, *NF* neurofilament, *Pioglit* pioglitazone

### Animal behaviour

Both wild type mice and COX10Adv mutant mice of C57Bl6 background were subjected to no more than two behavioral tests, following a period of training. Animal in the survival experiment that reached a moderate severity as defined in the project license were culled using a Schedule 1 method.

### Electron microscopy of mouse spinal cord

Mice were euthanized using an overdose of pentobarbitone (200 mg/ml) and perfused intravascularly with 2.5% glutaraldehyde and 4% paraformaldehyde in 0.1 M sodium cacodylate buffer. Following post fixing of the spinal cord, tissue was prepared, embedded in Durcupan resin and stained, as previously described [63]. A JEOL JEM-1400 Plus transmission electron microscope was used with Gatan one view digital camera and Digital Micrograph 3 software at × 5700 magnification to image dorsal column axons in cross section. To calculate the *g* ratio, the cross-sectional area of the axon, both including and excluding myelin ring, was determined using the freehand tool on ImageJ software, which enable the radius of the axon and radius of the axon plus myelin ring to be calculated to determine the g-ratio. At least 100 dorsal column axons in the gracile fasciculus in the thoracic spinal cord were included for each mouse.

### Synaptoneurosome preparation and measurement of Ca^2+^ fluorescence responses

To investigate whether COX10 deficiency or focal dorsal column demyelination impact on neurotransmission at the first central synapses of DRG neurons, we prepared synaptoneurosomes from dorsal column nuclei (DCN) [24, 73]. Protocols that optimise metabolic and ionic integrity in synaptoneurosomes have been recently developed in our laboratory so dynamic Ca^2+^ fluorescence responses to receptor stimuli can be measured [43, 68, 74]. The selective AMPA receptor agonist, R,S-AMPA (Abcam) with the selective inhibitor of AMPA receptor desensitization, cyclothiazide (Tocris) or the Ca^2+^ ionophore, ionomycin (Abcam), were added immediately before recording. Intracellular Ca^2+^ fluorescence was read at excitation 488 nm, emission 518 nm. Ionomycin (10 µM) and basal measurements were included in every plate to calibrate the dynamic range of the assay. Mean responses were calculated over the first 4 min following drug addition.

### Statistical analysis

GraphPad Prism^®^ software, version 6.0 (GraphPad Software Inc, US) was used for statistical analysis. For normally distributed data a two-way student’s *t* test was used to determine statistical significance, while One-way ANOVA was used for multiple column comparisons. For data that was not normally distributed the two-tailed ‘Mann–Whitney *U*’ test was selected as a non-parametric test, while multiple column comparisons were done by using the Kruskal–Wallis test. Unless otherwise specified, data is represented as the average ± standard deviation (SD). A *p* value < 0.05 was regarded as being significant and designated *^–^**** in the graphs.

## Results

### Mitochondria mobilise from the neuronal cell body to the axon, increasing mitochondrial content following demyelination: the axonal response of mitochondria to demyelination (ARMD)

We hypothesized that mitochondria within healthy neuronal cell bodies might respond to demyelination by moving to the axon. We therefore labeled mitochondria with a photoconvertible protein (mito-mEOS2) and performed live imaging over 20 min to visualise mitochondria that enter the myelinated and demyelinated axons from the cell body in cerebellar slices (Fig. 1a and Supplementary Fig. 1, online resource). Following photoconversion of axonal mitochondria, we identified a large increase in the number of unconverted mitochondria moving from the Purkinje cell body to the proximal axon segment upon demyelination (Fig. 1c, e–g and videos 6–10, online resource), compared to control (Fig. 1b, d, f–g and videos 1–5, online resource). Furthermore, the motile mitochondria displayed a greater anterograde speed in demyelinated axons compared with myelinated axons (Fig. 1h). These effects of demyelination on axonal mitochondria are not an artefact of the demyelinating agent, lysolecithin, since it did not significantly impact mitochondrial movement in *Shiverer* mice where axons lack myelin (Supplementary Fig. 2, online resource) [10, 28]. To assess this mitochondrial response from cell body to the demyelinated axon in another neuronal subtype, we cultured DRG neurons in the cell body compartment of microfluidic chambers, myelinated their axons in a separate chamber, induced demyelination by exposing axonal compartment to lysolecithin and again found evidence of increased mitochondrial mobilisation from the neuronal cell body to the axon and increased axonal mitochondrial content (Supplementary Fig. 3, online resource). We term this homeostatic response the “axonal response of mitochondria to demyelination” (ARMD).

### The homeostatic ARMD is not sufficient to protect acutely demyelinated axons from degeneration

Because the increased transport of mitochondria from the neuronal cell body to the axon upon demyelination requires time to build up axon mitochondrial content in the demyelinated axon we quantitated changes in axonal mitochondrial content over time. ARMD peaked at 5 days post demyelination in cerebellar slices and at 7 days post demyelination of centrally projecting dorsal column axons of DRG neurons, in vivo. To examine the impact of ARMD on axonal health, we compared the temporal changes of axonal degeneration, indicated by the formation of axonal bulbs [70], with that of axonal mitochondrial content (Fig. 1i–l). In both demyelinated cerebellar slices and the centrally projecting dorsal column axons of DRG neurons in vivo, transection of Purkinje cell and DRG axons occurred 2–4 days prior to the peak of ARMD (Fig. 1i–l). We conclude that the acutely demyelinated axon is particularly vulnerable for at least 2–4 days, until ARMD reaches its peak, signifying a potentially short therapeutic window for neuroprotection.

### Targeting mitochondrial dynamics and biogenesis increases the mobilisation of mitochondria from neuronal cell body to the axon

Given that the movement of mitochondria from the neuronal cell body to increase the mitochondrial content of the acutely demyelinated axon is a relatively protracted process compared with the rapid degeneration of the axon, we aimed to determine whether the influx of mitochondria from the cell body to the axon can be increased. We first over-expressed Mitochondrial Rho GTPase1 (Miro1), which is known to facilitate mitochondrial transport by tethering mitochondria to a motor/adaptor protein complex [22], and found that this significantly increased the movement of mitochondria from the cell body to the axon in unmyelinated DRG neurons (Fig. 2a, b), as expected [61]. However, targeting mitochondrial transport alone did not significantly affect the total mitochondrial content of the axons, due to the large number of stationary mitochondria present there (Fig. 2biv–v). Therefore, we aimed to stimulate mitochondrial biogenesis in neurons by over-expressing the peroxisome proliferator-activated receptor gamma (PPAR-γ) coactivator 1-alpha (PGC1α), which is the master regulator of mitochondrial biogenesis (Fig. 2c) [44, 75]. Over-expression of PGC1α in DRG neurons led to increased anterograde mitochondrial transport and increased mitochondrial axonal content, thus mimicking ARMD. The result of the genetic manipulation was recapitulated upon pharmacological application of pioglitazone, an established PGC1α pathway agonist (Fig. 2d) [44]. These findings were specific to mitochondria, since lysosomal transport was unaffected (Fig. 2e). We next examined mitochondrial content in myelinated axons following over-expression of Miro1, PGC1α and application of pioglitazone, and found similar changes to unmyelinated axons (Fig. 2h–j). Taken together, these findings indicate the potential of targeting mitochondrial dynamics and biogenesis in neurons to enhance ARMD.

### Promoting ARMD in healthy neurons protects the acutely demyelinated axons from degeneration

We next assessed whether enhancing the mobilisation of mitochondria from the neuronal cell body to the axon alone or in combination with increased biogenesis could protect the acutely demyelinated axons from degeneration in three experimental systems. First, we found that the over-expression of Miro1, PGC1α, or the application of pioglitazone specifically to the DRG neuronal cell bodies in microfluidic chambers, significantly decreased the fragmentation of acutely demyelinated axons and significantly increased the number of intact demyelinated axons (Fig. 3a–c). Addition of a PGC1α inhibitor to the neuronal cell body chamber together with pioglitazone reversed the protective effect of pioglitazone treatment on demyelinated axons, implicating PGC1α pathway in pioglitazone induced neuroprotection (Fig. 3c). We also applied pioglitazone to cerebellar slice cultures and noted a significant increase in the axonal mitochondrial content and a significant decrease in axonal degeneration following demyelination (Fig. 3d–f). We next administered pioglitazone to wild type mice for 6 weeks prior to focal demyelination of the dorsal columns and found a significantly decreased axonal bulb formation with treatment (Fig. 3j–l). Given the pleiotropic effects of pioglitazone, we investigated whether expression of its target, PGC1α, was affected in DRG neurons and found a significant increase of PGC1α + nuclei in DRG neurons with treatment (Supplementary Fig. 4, online resource). In keeping with in vitro findings in DRG neurons following over expression of PGC1α and pioglitazone treatment, we found a significant increase in mitochondria content within axons, in vivo, with pioglitazone treatment (Fig. 3m). Taken together, these in vitro and in vivo findings indicate that the increased mobilisation of mitochondria from the neuronal cell body to the axon by targeting of PGC1α pathway and over-expression of Miro1 enhances ARMD in wild type neurons and protects acutely demyelinated axons from degeneration.

### Dorsal root ganglia neurons in progressive multiple sclerosis display complex IV deficiency and an increase in mitochondrial content in demyelinated axons

In disease states, such as progressive MS, there is perturbation to the function of mitochondria in neurons, exemplified by complex IV deficiency, the terminal complex of the electron transport chain. Complex IV deficiency is known to impair anterograde mitochondrial transport in myelinated axons and deplete the mitochondrial content of myelinated axons [6, 9, 14, 32, 33, 53, 78]. This raises the question whether mitochondria in these complex IV deficient neurons in disease states can respond to demyelination. Therefore, we wanted to test the relevance of ARMD to demyelinating diseases with complex IV deficient neurons, by examining respiratory deficient DRG neurons in MS autopsy tissue and the mitochondrial parameters of their demyelinated axons at the dorsal root entry zone [6, 9, 14, 78].

**Fig. 4:**
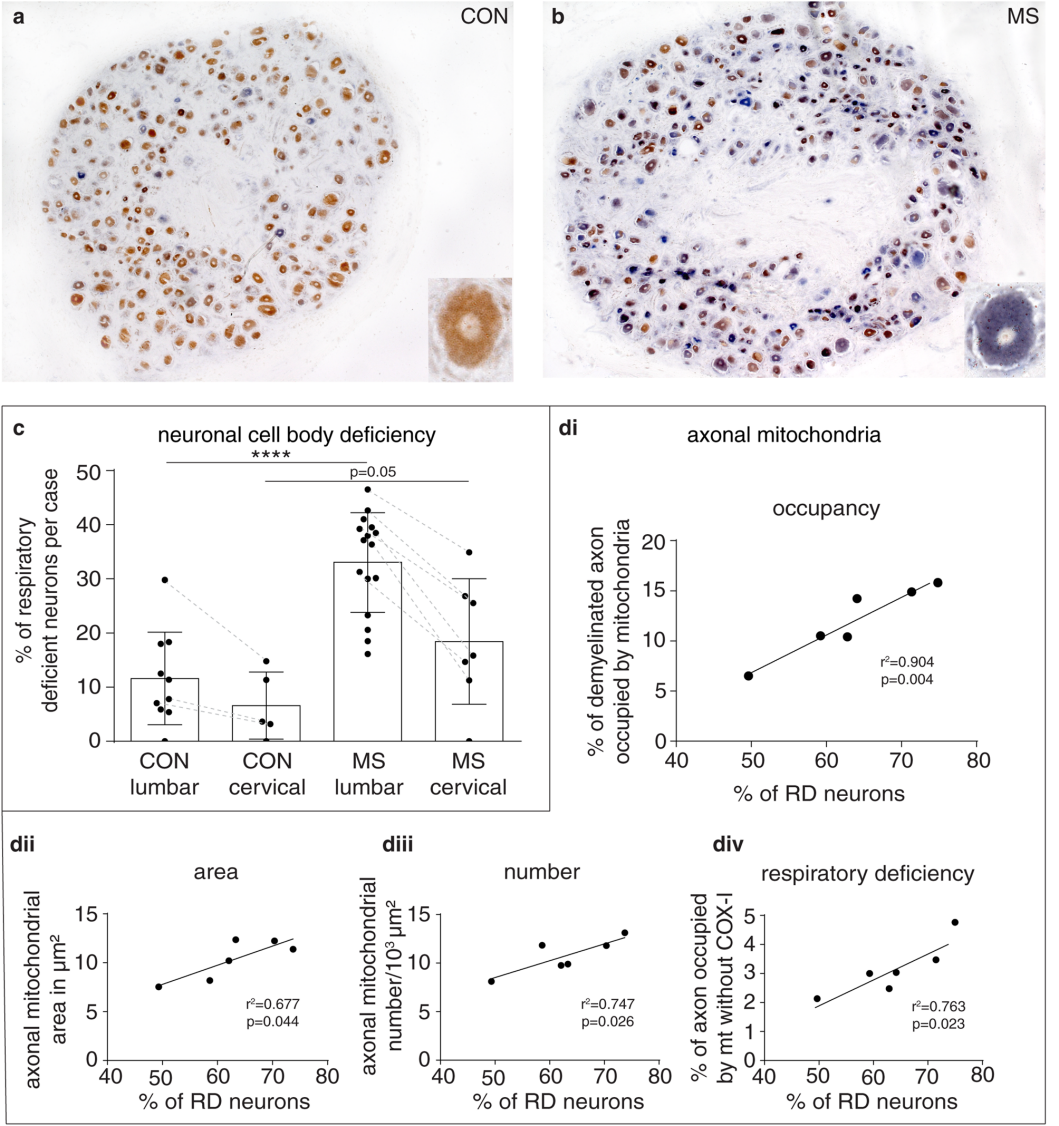
Respiratory deficient neurons are prevalent within dorsal root ganglia in progressive MS and their percentage positively correlates with mitochondrial content, size, number and complex IV deficiency in demyelinated dorsal column axons. **a, b** In progressive MS, dorsal root ganglia (DRG) neurons that lack mitochondrial complex IV and contain complex II (stained blue by COX/SDH histochemistry, insert in **b**), termed respiratory deficient, are abundant (**b**) compared with controls (**a**). The majority of neurons show intact complex IV in controls (stained brown, insert in **a**). **c** Quantitation of DRG in progressive MS identified significantly more respiratory deficient neurons in lumbar DRGs compared with controls (*p* < 0.0001). Respiratory deficient neurons tended to be more prevalent in lumbar DRG than cervical DRG [the broken lines (**c**) indicate data from the same case]. In Parkinson’s disease the mean respiratory deficient neurons in lumbar DRG is 28.77% (SD = 13.66, *n* = 3) and the mean is 33.51% (SD = 13.66, *n* = 2) in motor neuron disease (MND) lumbar DRG (not shown). Data presented as dot-plot with mean (bar) ± standard deviation (whiskers). ****p < 0.0001 using Mann–Whitney *U* test and Kruskal–Wallis test showed a *p* < 0.0001 in multiple subgroup comparisons. Chronic spinal cord lesions in dorsal columns, at the corresponding dorsal root entry zone, of six progressive MS cases enabled the impact of respiratory deficient neurons on the mitochondrial parameters of demyelinated axons to be assessed. **d** Mitochondrial content in demyelinated dorsal column axons at the root entry zone, as a percentage of axon area, correlated significantly (*p* = 0.004, *r*^2^ = 0.904) and positively with the percentage of respiratory deficient proprioceptive neurons in lumbar DRG in progressive MS (**d**i). Average size of mitochondria within demyelinated dorsal column axons correlated significantly (*p* = 0.044, *r*^2^ = 0.677) and positively with the percentage of respiratory deficient proprioceptive neurons in lumbar DRG in progressive MS (**d**ii). Average number of mitochondria within demyelinated dorsal column axons at the root entry zone correlated significantly (*p* = 0.026, *r*^2^ = 0.747) and positively with the percentage of respiratory deficient proprioceptive neurons in lumbar DRG in progressive MS (**d**iii). As expected, the percentage of the axon occupied by mitochondria that lacked complex IV subunit-I correlated significantly (*p* = 0.023, *r*^2^ = 0.763) with the percentage of respiratory deficient proprioceptive neurons in lumbar DRG in progressive MS (**d**iv)

We studied DRG neuronal cell bodies and their demyelinated centrally projecting axons in the spinal cord dorsal columns in progressive MS, as this enabled us to accurately identify mitochondria in cell bodies and associated demyelinated axons (Table 1). We found approximately one third of neuronal cell bodies in DRG of MS to be complex IV deficient (Fig. 4a–c) due to clonally expanded mitochondrial DNA deletions (Supplementary Fig. 5, online resource). Histological analysis of DRG in MS revealed a significant increase in the number of HLA + and GFAP + cells while the neuronal cell body count did not differ significantly between MS and controls, indicating a reactive milieu (Supplementary Fig. 6, online resource). In 6 out of 18 progressive MS cases, we identified chronically demyelinated axons in dorsal columns at the dorsal root entry zone (Fig. 4d), and found positive correlations between the percentage of complex IV deficient proprioceptive neuronal cell bodies in the DRG and the mitochondrial content, mitochondrial area, mitochondrial number and impaired complex IV in associated demyelinated dorsal column axons (Fig. 4d). This statistical association indicates that complex IV deficient neuronal cell bodies, harboring clonally expanded mtDNA deletion, respond to demyelination by mobilising mitochondria to the axon, despite their respiratory deficiency.

### Existing animal models of MS lack complex IV deficient neurons and complex IV knockout mice exhibit the ARMD

**Fig. 5:**
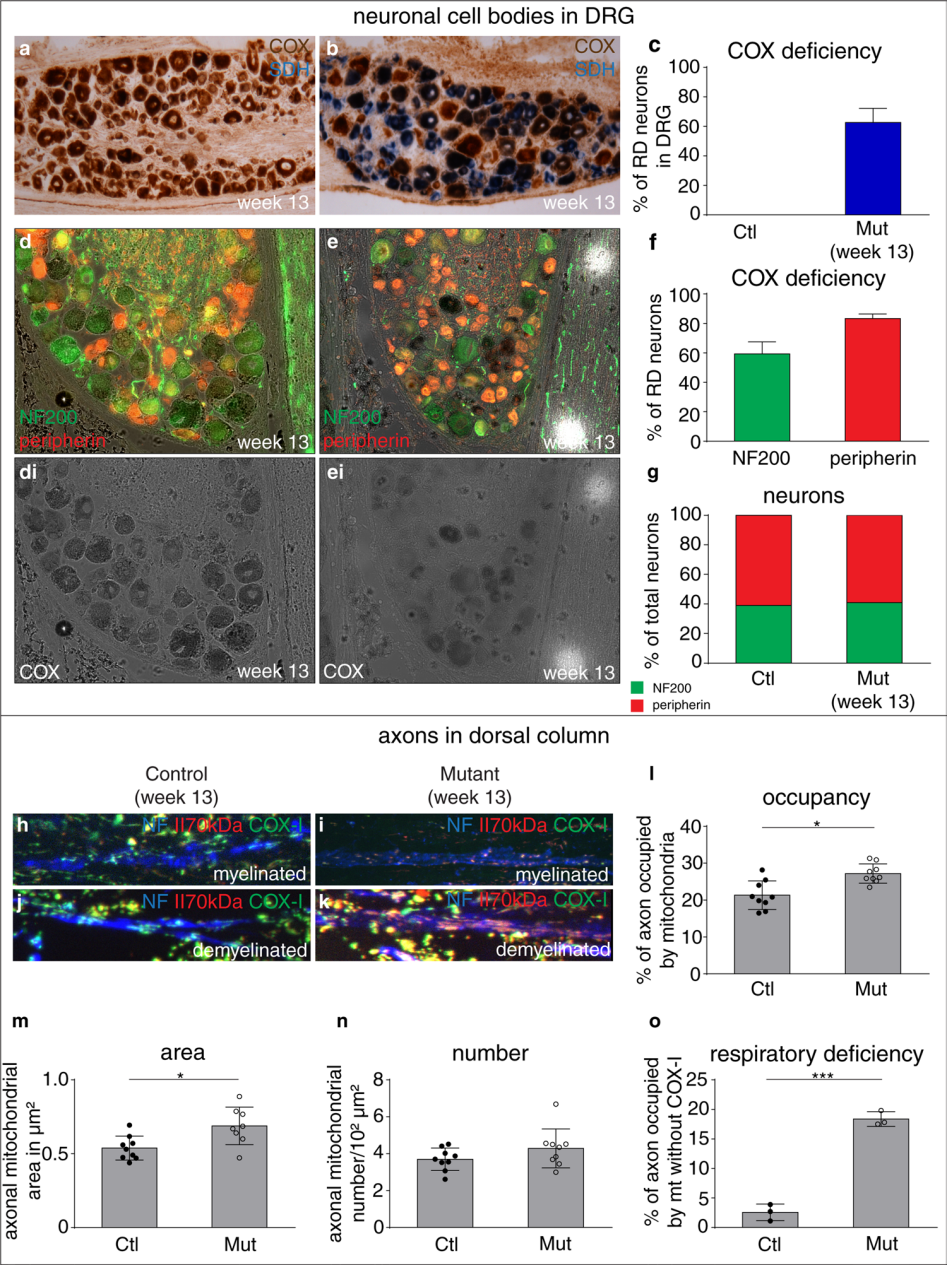
Modeling the complex IV deficient DRG neurons and recapitulating mitochondrial changes within demyelinated axons in progressive MS, in vivo. **A–c** DRG neuron-specific inducible knockout mice that lack protoheme IX farnesyltransferase [subunit 10 of complex IV (COX10), termed COX10Adv mutants) contain complex IV deficient DRG neurons (**b**), which are stained blue by the sequential COX/SDH histochemistry assay. DRG neurons with intact complex IV are stained brown in both wild type mice and COX10Adv mutants (**a**, **b**). The quantitation identified approximately 59% of the DRG neurons as respiratory deficient in COX10Adv mice (**c**, *n* = 3) compared with none in controls (**c**, *n* = 3). **d–f** Sequential COX histochemistry and immunofluorescent labeling method, as previously described, identifies both proprioceptive (NF200 + peripherin-, in green) and nociceptive neurons (NF200-peripherin + , in red) in DRG that are respiratory deficient (lack of or decrease in intensity of brown staining following COX histochemistry, shown in grey scale images in **d**i and **e**i) in COX10Adv mutants (**e**i), unlike wild type mice (**d**i). The quantitation of complex IV within immunofluorescently labeled proprioceptive and nociceptive neurons, using densitometry, identifies approximately 59.33 ± 8.14% and 83.33 ± 3.05% of proprioceptive and nociceptive neurons, respectively, in COX10Adv mutants to be lacking complex IV (**f**). Complex IV deficient neurons, judged by densitometric analysis of COX histochemistry, are not present within DRG from wild type mice (**a**). **g**: Quantitation of proprioceptive (NF200-positive in green) and nociceptive (peripherin-positive in red) DRG neurons from 13 week old wild type (*n* = 3) and COX10Adv mutants (*n* = 3) shows similar proportion of proprioceptive and nociceptive neurons in both groups. **h–k** In focal lysolecithin-induced lesions of the dorsal columns, demyelinated axons (NF in blue) contain more mitochondria (**j**, **k**) than myelinated axons (**h**, **i**), when mitochondria are identified with complex II 70KDa subunit (red) labeling, in both wild type (**h**, **j**) and COX10Adv mutants (**i**, **k**). As expected, complex IV subunit-I (green) is lacking within axonal mitochondria in COX10Adv mutants (**i**, **k**). **l–o** Quantitation of mitochondria within demyelinated dorsal column axons from wild type mice and COX10Adv mutants shows a significantly greater mitochondrial occupancy (**l**) and mitochondrial size (**m**) as well as extent of mitochondria lacking complex IV subunit-I (**o**) in experimentally demyelinated COX10Adv mutants compared wild type mice. These findings are concordant with the positive correlations that we observed between the extent of respiratory deficient proprioceptive neurons and the mitochondrial parameters within demyelinated dorsal column axons at the root entry zone in progressive MS (shown in **e**, **f**). *p < 0.05 and ***p < 0.001 using Mann–Whitney *U* test. Data presented as dot-plot with mean (bar) ± standard deviation (whiskers)

To investigate ARMD in complex IV deficient neurons, we studied 9 distinct experimental models of demyelination that are pertinent to MS and a model of traumatic axonal transection (Table 2). However, we did not find complex IV deficient neurons within the brain, spinal cord or DRG in any of these models (Supplementary Fig. 7, online resource) and mitochondrial DNA deletions were rarely detected in any of these animal models (Supplementary Fig. 8, online resource). Therefore, to model the complex IV deficient DRG neurons that are observed in MS, we developed a neuron-specific inducible mitochondrial mutant by knocking out complex IV subunit 10 (COX10 or protohaem IX farnesyltransferase) in DRG neurons (COX10Adv mutant mice, Fig. 5) [13, 31]. In 13 week old COX10Adv mutants, 59% of proprioceptive DRG neurons were complex IV deficient (Fig. 5c), but showed no signs of behavioral disruption or neurodegeneration (Supplementary Fig. 9 + 10, online resource). Similar to the observations in MS autopsy tissue, we found that mitochondrial content, area and complex IV deficiency was significantly increased in demyelinated axons in COX10Adv mutant mice (Fig. 5l–o). Mitochondrial content of myelinated axons did not significantly differ in COX10Adv mutant mice compared with controls (Supplementary Fig. 11, online resource). The fact that the ARMD occurs in complex IV deficient neurons in an experimental model provided us with the opportunity to test whether enhancing ARMD can protect these demyelinated axons, which are acutely vulnerable to degeneration.

### Promoting ARMD in complex IV deficient neurons is neuroprotective

**Fig. 6:**
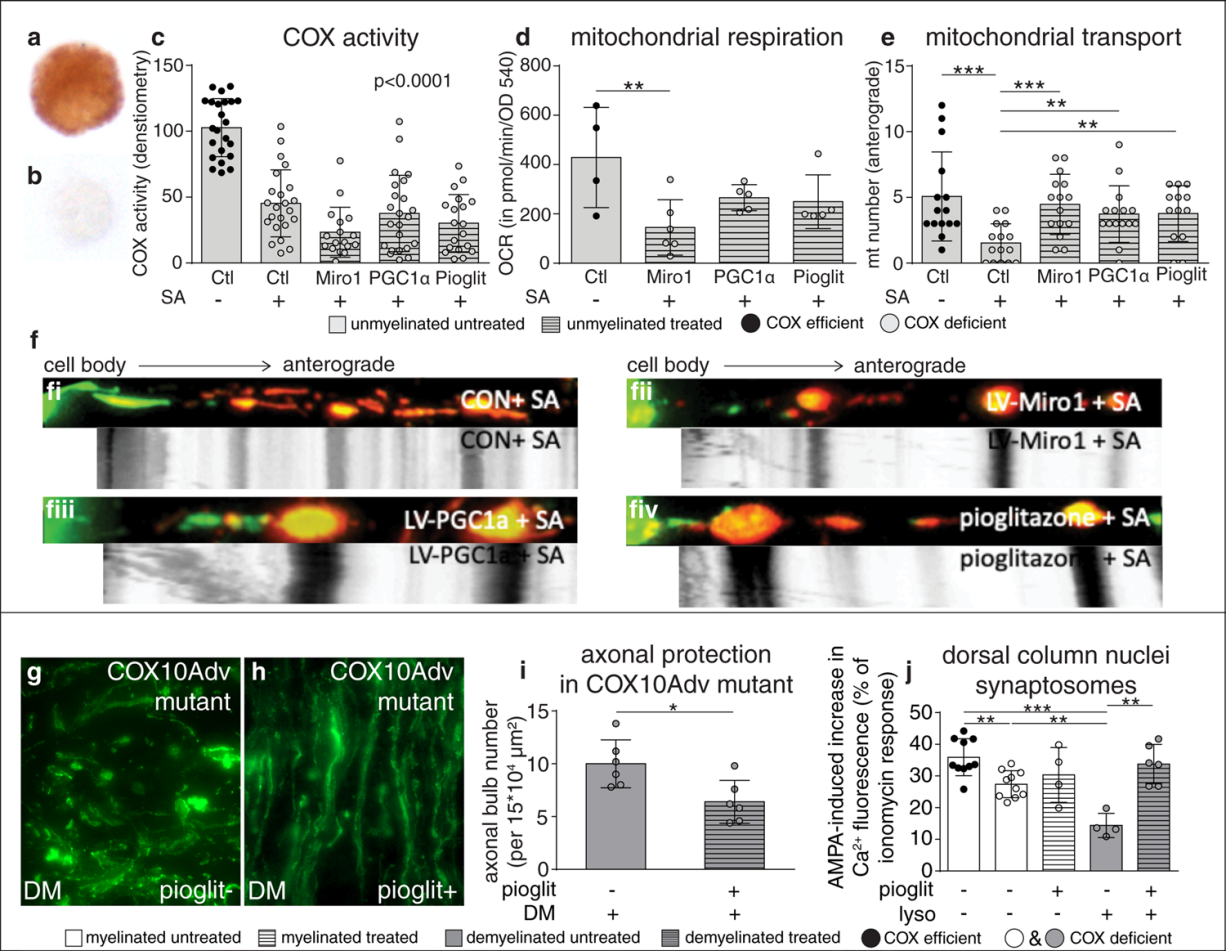
Enhancement of ARMD in complex IV deficient neurons protects the extremely vulnerable acutely demyelinated axons. **a–c** To model complex IV deficiency in vitro, we pharmacologically inhibited it using sodium azide (SA, at 100 μM for 16 h), which significantly decreases complex IV activity in wild type DRG neurons (**b**, **c**), as expected, compared with controls (**a**, **c**). The inhibition of complex IV by SA is similarly effective in DRG neurons, where Miro1 and PGC1α are over-expressed, using lentiviruses, and when exposed to pioglitazone (**c**). Controls shown were exposed to lentivirus-mEOS2. Solvent (DMSO) only controls for pioglitazone treatment did not show a significant effect compared with lentivirus-mEOS2 controls without DMSO (not shown). ***p < 0.001 using Mann–Whitney *U* test and Kruskal–Wallis test showed a *p* < 0.05 in multiple subgroup comparisons. **d** Mitochondrial respiration significantly decreases when DRG neurons that are over-expressing Miro1 are exposed to SA (100 μM for 16 h) (**d**). Although mitochondrial respiration tends to decrease when DRG neurons that are over-expressing PGC1α and treated with pioglitazone are exposed to SA, the decrease is not statistically significant (**d**), despite the significant inhibition of complex IV activity (**c**). **p < 0.01 using Mann–Whitney *U* test and Kruskal–Wallis test showed a *p* < 0.05 in multiple subgroup comparisons. **e, f** As expected, the number of mitochondria mobilising from the DRG neuronal cell body to the axon significantly decreases following inhibition of complex IV by SA (100 μM for 16 h) compared with DRG neurons not exposed to SA. In contrast, SA does not significantly decrease the anterograde movement of mitochondria from the cell body to the axon in DRG neurons where Miro1 and PGC1α are over-expressed, using lentiviruses, and when DRG neurons are treated with pioglitazone (**e**). Kymographs show the improvement in the number of mitochondria mobilising from the complex IV deficient neuronal cell body to the axon, which is mediated by Miro1 (**f**ii), PGC1α (**f**iii) and pioglitazone (**f**iv). **p < 0.01 and ***p < 0.001 using Mann–Whitney *U* test and Kruskal–Wallis test showed a *p* < 0.05 in multiple subgroup comparisons. **g–i** When dorsal column axons are demyelinated (**g**, DM) by focal lysolecithin injections to the dorsal columns of COX10Adv mutant mice with complex IV deficient DRG neurons, there is an abundance of axon bulbs at 3 days post lesioning (**g**, **i**). Pioglitazone in diet for 6 weeks (120 mg/kg diet), prior to focal dorsal column demyelination, significantly decreased the axon bulb formation within the demyelinated area in COX10Adv mutant mice (**h**, DM pioglit +) compared with untreated COX10Adv mutants (**g**, DM pioglit-) after focal demyelination of dorsal columns (**i**). *p < 0.05 and **p < 0.01 using Mann–Whitney *U* test. **j** Ca^2+^ fluorescence responses of dorsal column nuclei (DCN) synaptoneurosomes evoked by AMPA (40 μM) in the presence of cyclothiazide (20 μM) were significantly attenuated in COX10Adv mutant mice compared to wild type and the impairment was exacerbated in mice with dorsal column demyelination 3 days previously. Dietary treatment with pioglitazone for 6 weeks beforehand fully rescued the functional deficits. Responses of non-pioglitazone controls (treated with DMSO vehicle) showed no significant difference from naive controls (**p < 0.01, ***p < 0.001; One-Way ANOVA with Tukey’s test, *n* = 4–10). COX10Adv: Inducible and DRG neuron-specific (Adv: advillin) knock out of complex IV subunit 10 (COX10) in mice, with complex IV deficient DRG neurons. Data presented as dot-plot with mean (bar) ± standard deviation (whiskers). *DM* demyelinated, *Pioglit* pioglitazone, *SA* sodium azide, complex IV inhibitor. *p < 0.05, **p < 0.01 and ***p < 0.001

Given that complex IV deficient neurons demonstrate ARMD, we tested whether transport of mitochondria from complex IV deficient neuronal cell bodies to the axon can be enhanced by targeting mitochondrial over-expression of Miro1, PGC1α and pioglitazone treatment. We inhibited complex IV and therefore mitochondrial respiration in mature DRG neurons, in vitro*,* by using sodium azide (SA), which resulted in a significantly decreased anterograde transport of mitochondria, as expected (Fig. 6a–c) [39]. Strikingly, over-expression of Miro1 or PGC1α and treatment with pioglitazone overcame the anterograde mitochondrial transport deficit present in complex IV deficient neurons (Fig. 6e, f). Furthermore, targeting mitochondrial biogenesis with PGC1α over-expression and pioglitazone treatment limited the SA induced inhibition of mitochondrial respiration, presumably due to the increased mitochondrial content in neurons (Fig. 6d and Supplementary Fig. 12, online resource) [44]. Finally, we tested whether enhancing the ARMD in complex IV deficient neurons may also be neuroprotective, in vivo, in the context of demyelination. Consistent with our in vitro findings, the treatment of COX10Adv mutant mice with dietary pioglitazone for 6 weeks significantly increased axonal mitochondrial content and the percentage of proprioceptive neurons with PGC1α-positive nuclei (Supplementary Fig. 13, online resource). Dietary pioglitazone neither change the number of nuclei, identified by DAPI staining, nor the number of Iba1 positive microglia in focal lesions, although there was a trend towards decreasing Iba1 positive microglia with treatment (Supplementary Fig. 14, online resource) [46, 66]. Strikingly, the increased mobilisation of mitochondria in complex IV deficient neurons and axonal mitochondrial content, by pioglitazone treatment, protected the acutely demyelinated axons as evident by the significant decrease in acutely transected axons in demyelinated lesions (Fig. 6g–i).

To determine whether the protection of acutely demyelinated axons in COX10Adv mutant mice affects functional connectivity, we evaluated the excitability of the first central synapses of the dorsal column axons in the dorsal column nuclei (DCN). We isolated functionally intact synapses from DCN, using synaptoneurosomal preparations, and exposed to AMPA receptor agonists to assess Ca^2+^ responses. AMPA-induced Ca^2+^ fluorescence responses of freshly prepared DCN synaptoneurosomes were significantly reduced in COX10Adv mutant mice compared to wild type controls and the deficit was exacerbated 3 days following dorsal column demyelination (Fig. 6j). Importantly, pioglitazone treatment significantly improved the excitability of DCN synaptoneurosome derived from the complex IV deficient neurons that were experimentally demyelinated (Fig. 6j). Thus, pioglitazone treatment protects not only the structural integrity of acutely demyelinated axons in COX10Adv mutant mice, but also downstream synaptic function.

## Discussion

**Fig. 7:**
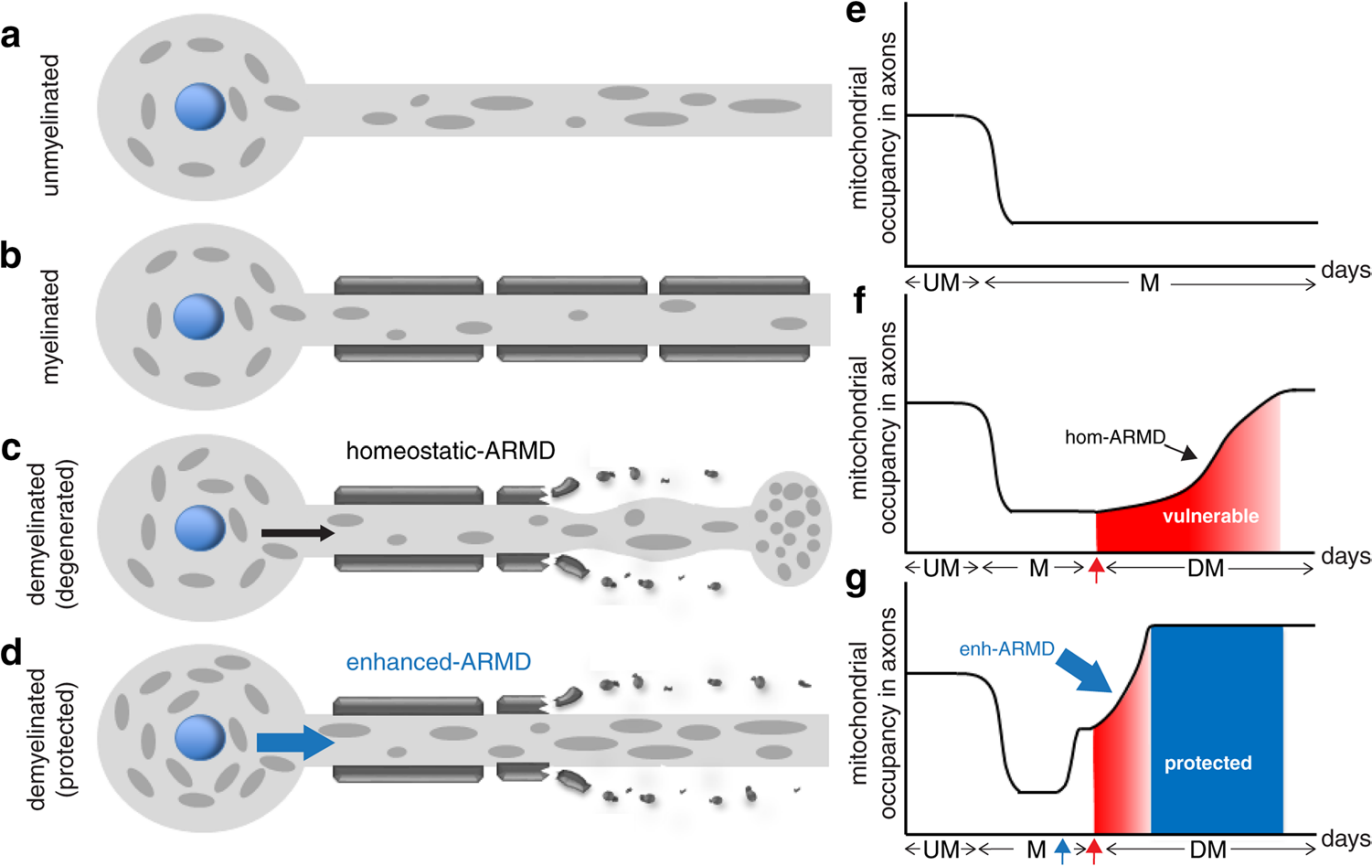
Schematic of the novel neuroprotective strategy to preserve acutely demyelinated axons by increasing the mobilisation of mitochondria from the neuronal cell body to the axon. **a–g** Energy efficiency offered by myelination is reflected by a decrease in mitochondrial content in myelinated axons compared with unmyelinated axons, which is elegantly illustrated by the healthy optic nerve (**a**, **b**) [5]. Immediately following myelin loss, we show that mitochondria increasingly mobilise from the neuronal cell body to the acutely demyelinated axons, leading to a gradual increase in the axonal mitochondrial content (**c**, **f**), which we term axonal response of mitochondria to demyelination (ARMD). ARMD is a homeostatic phenomenon that attempts to increase the energy producing capacity of the acutely demyelinated axons (hom-ARMD). However, hom-ARMD is not sufficient to protect the acutely demyelinated axon, which undergoes transection within days of myelin loss and where myelin debris is still evident (**c**). During the time that is required by hom-ARMD to increase the mitochondrial content of the demyelinated axons (days), towards the level of the unmyelinated axons, the acutely demyelinated axons are particularly vulnerable and degenerate (**c**, **f**). Increased mobilisation of mitochondria from the neuronal cell body to the axons, which results in an enhanced ARMD (enh-ARMD) in both wild type neurons and those with complex IV deficiency, protects the vulnerable acutely demyelinated axons (**d**, **g**). This novel neuroprotective strategy is crucial to protect the acutely demyelinated axons so that, subsequently, neurorestorative therapy like remyelination can be implemented in demyelinating disorders. *ARMD* axonal response of mitochondria to demyelination, *DM* demyelinated axon, *Enh-ARMD* enhanced ARMD, *Hom-ARMD* homeostatic ARMD, *M* myelinated axon, *UM* unmyelinated axon

We propose a novel model (Fig. 7) based on our finding that demyelination per se creates a relative shortfall in the energy producing capacity, through the inability of the axon to rapidly increase its mitochondrial content; thus, the axon is not able to meet the increased energy demand that follows the loss of myelin. Myelination is associated with a decrease in axonal mitochondrial content, as evidenced in myelinated optic nerve axons and unmyelinated axonal segments in lamina cribrosa as well as dysmyelinated axons in *Shiverer* mice (Fig. 7a–b, e) [3, 5]. Upon demyelination, we discovered that mitochondria mobilise from neuronal cell body to the acutely demyelinated axons, which slowly build up their mitochondrial content through ARMD (Fig. 7b–c, f). Although the neuronal cell body attempts to energetically support the demyelinated axon, by increasing the transport of mitochondria to the axon, the peak of ARMD lags behind the peak of axon transection by a number of days. Thus, the homeostatic response of ARMD is insufficient to correct the resulting energy imbalance created by demyelination (Fig. 7f). By targeting mitochondrial biogenesis and anterograde transport to enhance ARMD, we identify a novel therapeutic strategy to protect the acutely demyelinated axons (Fig. 7c–d, g).

Currently, there is no effective neuroprotective therapy for demyelinating disorders, including MS. One strategy is to curtail the increased energy demand of demyelinated axon through drugs that inhibit sodium channels. Although effective in experimental models, these have failed in clinical trials and are poorly tolerated by MS patients [27, 76]. This is perhaps not surprising because they perturb adaptive neuronal firing properties that are required for healthy functioning. An alternative strategy, which we show here, is to boost the energy producing capacity of the demyelinated axon. Previous studies have shown that axonal mitochondrial transport can be increased by targeting mitochondrial biogenesis, through PGC1α over expression, as a potential therapy for neurodegenerative disorders [50].This potential therapeutic strategy, however, has not been applied to demyelinating disorders. We show that an increased transport of mitochondria from the neuronal cell body to axon makes ARMD more efficient and protects the acutely demyelinated axon. Given the suboptimal nature of the homeostatic ARMD, the strategy of increasing mitochondrial biogenesis and axonal transport is ideally suited for demyelinating disorders. Hence, our findings provide a mechanism that can be therapeutically targeted—within the necessary short time frame—for neuroprotection in demyelinating disorders.

Notwithstanding the prevalence of sensory symptoms, including impaired joint position sensation and increased sensitivity to painful stimuli in MS, coupled with the prediction of metabolic and molecular perturbations in the MS DRG, it is surprising that DRG neurons have not been hitherto studied [69]. We performed a detailed analysis of mitochondria in DRG neuronal cell bodies from 18 progressive MS autopsy cases and 12 controls. Furthermore, we correlated mitochondrial changes within DRG neuronal cell bodies with mitochondrial changes within demyelinated axons, at the dorsal root entry zone, in spinal cord blocks. Complex IV deficiency and clonally expanded mtDNA deletions in DRG neurons, as observed in this study, are similar to those seen in cortical neurons and choroid plexus epithelial cells in MS, although the respiratory deficiency affected a greater proportion of neurons in the DRG [7, 9]. These findings show that there are factors other than the energy shortfall due to the time lag of ARMD and inflammation induced damage to mitochondria, which are intrinsic to the neuron that contribute to the energy deficit of the demyelinated axon. MtDNA deletion in single cells is not a reflection of the accumulation of ongoing damage to mtDNA. Clonal expansion of mtDNA is an active phenomenon that amplifies a mtDNA deletion in a single cell, as evidenced in a number of neurodegenerative disorders, including MS [29]. In MS, mtDNA deletions appear to be induced by the inflammatory process, as we did not find changes, in excess of age, in skeletal muscle from progressive MS cases. These mtDNA deletions then undergo amplification through clonal expansion in metabolically highly active cells such as neurons and choroid plexus epithelial cells [8]. Given the clonally expanded mtDNA deletions in DRG neurons, together with the significant positive correlation between the extent of complex IV deficiency in proprioceptive DRG neuronal cell bodies and axonal mitochondrial content, we suggest that complex IV deficient neurons attempt to trigger ARMD even more vigorously than neurons with healthy mitochondria.

The failure to identify respiratory deficient neurons in the brain, spinal cord and DRG following a detailed examination in nine experimental disease models, and tissue from spinal cord hemi-section, highlights a major shortfall of the existing disease models in recapitulating neuropathological findings of progressive MS. There are multiple potential reasons for the lack of respiratory deficient neurons in existing models. First, oxidative injury that is implicated in the induction of mtDNA deletions is limited in established disease models compared with MS [60]. Second, these experimental disease models predominantly adopt young animals, which may have better repair and mitochondrial quality control mechanisms. Third, the clinicopathological course of animal models is relatively short compared with the disease duration of progressive MS. Finally, both chronic and ongoing acute demyelination, as evidenced in slowly expanding MS lesions, are relatively sparse in existing disease models compared with progressive MS [19]. Although the complex IV knockout mice used in this study do not elucidate the causes of mitochondrial perturbations in neuronal cell bodies, such models recapitulate the neuronal mitochondrial changes in MS and therefore help to determine the consequences of mitochondrial respiratory chain deficiency for the demyelinated axons. Our data in COX10Adv mutants indicate that the stimulation of mitochondrial biogenesis and mitochondrial dynamics can partly overcome the detrimental consequences of the complex IV deficiency in acutely demyelinated axons. The protective effect of increasing mitochondrial biogenesis in complex IV deficient neurons is likely to reflect the fact that such mitochondria are deficient, but not completely devoid of metabolic activity. Therefore, despite their deficiency, they exert a net positive effect, and help boost the overall energy producing capacity of the axon. We therefore suggest that enhancing ARMD is a therapeutically tractable approach, particularly when combined with approved therapy in MS as well as HIV neuropathy and diabetic neuropathy, where complex IV deficiency is an additional contributor to the axonal energy failure [6, 9, 14, 78].

In demyelinating disorders, axon degeneration is most prominent in areas with acute demyelination. In MS, neuropathological studies have shown that demyelination is ongoing throughout the clinical course of the disease. During the early stage of MS, there is an abundant formation of acutely demyelinating lesions [19]. In progressive MS, there is still ongoing demyelination, particularly at the edge of chronic active or slowly expanding lesions even at the end stages of MS [19, 35]. MRI imaging provides robust evidence of new lesions in early stage and slowly expanding lesions [1, 11, 19]. In terms of neuronal mitochondria, factors that amplify mitochondrial injury, such as oxidative stress and iron accumulation, likely require time to compromise the neurons [37]. Our prediction is that respiratory deficiency within neurons becomes more prominent with increasing disease duration in MS. Thus, our findings in both wild type mice and COX10Adv mutant mice show that our neuroprotective strategy is applicable to the entire disease course of MS.

Overall, for a neuroprotective strategy such as enhancing ARMD to be effective, it has to be combined with effective immunomodulatory therapies and with those that restore the myelin sheath to axons (remyelination therapy) [18]. The mechanisms of axon degeneration, such as by cytotoxic T cells mediated axonal transection and by free radical mediated damage to mitochondria in both myelinated and demyelinated axons require the inflammatory response in MS to be effectively controlled [23, 30, 49, 65]. Whether enhancing ARMD protects chronically demyelinated axons that are located in chronic inactive lesions or in the inactive centre of chronic active lesions is not known. Furthermore, whether the supply of metabolic substrate to the axon, that is necessary for mitochondrial respiratory chain function, is a rate limiting factor in both acutely and chronically demyelinated axons is not known [20]. The energy imbalance in chronically demyelinated axons can be partially restored by remyelination, which decreases the axonal mitochondrial content to a level that approaches that found in myelinated axons [81]. Remyelination addresses the long term protection of axons that have survived the acute destruction of their myelin sheath (chronically demyelinated axons). In contrast, our neuroprotection strategy allows more axons to be saved during acute demyelination so that remyelination may restore the metabolic neuronal-glial cross talk in the long term [1, 18, 20]. Thus, our neuroprotective model serves to bridge the crucial gap between immune therapy and regenerative therapy.

In summary, our findings clearly illustrate a key compensatory role for mitochondria as part of the neuronal response to demyelination. Although the mobilisation of mitochondria from the neuronal cell body to the axon occurs spontaneously following the destruction of myelin, the resultant increase in the mitochondrial content of the demyelinated axon, which we term homeostatic ARMD, is too protracted. Enhancing ARMD, by increasing the transport of mitochondria from the neuronal cell body to the axon as well as mitochondrial biogenesis in the neuron, protects the acutely demyelinated axon. This novel neuroprotective strategy is likely to be applicable to all demyelinating disorders, even when neurons are respiratory chain deficient. Hence, drugs that enhance ARMD are important to protect the vulnerable acutely demyelinated axons, so that regenerative strategies, like remyelination, can be effectively implemented in demyelinating CNS and PNS disorders.

## Electronic supplementary material

Below is the link to the electronic supplementary material.

Supplementary file1 (AVI 5836 kb)

Supplementary file2 (AVI 192 kb)

Supplementary file3 (AVI 136 kb)

Supplementary file4 (AVI 138 kb)

Supplementary file5 (AVI 133 kb)

Supplementary file6 (AVI 171 kb)

Supplementary file7 (AVI 109 kb)

Supplementary file8 (AVI 108 kb)

Supplementary file9 (AVI 116 kb)

Supplementary file10 (AVI 118 kb)

Supplementary file11 (AVI 151 kb)

Supplementary file12 (DOCX 67162 kb)
