# Supplementary material for: Enhanced axonal response of mitochondria to demyelination offers neuroprotection: implications for multiple sclerosis
Source: Acta Neuropathol. 2020 Jun 22;140(2):143–67. doi: 10.1007/s00401-020-02179-x (PMC7360646; doi:10.1007/s00401-020-02179-x)
Supplement: Supplementary file 12 — Supplementary file12 (DOCX 67162 kb) [file 401_2020_2179_MOESM12_ESM.docx]

**Enhanced axonal response of mitochondria to demyelination offers neuroprotection: implications for multiple sclerosis**

Simon Licht-Mayer†^1^, Graham R. Campbell†^1^, Marco Canizares†^1^, Arpan R. Mehta^1,2^, Angus B. Gane^1^, Katie McGill^1^, Aniket Ghosh^1^, Alexander Fullerton^1^, Niels Menezes^1^, Jasmine Dean^1^, Jordon Dunham^3^, Sarah Al-Azki^4^, Gareth Pryce^4^, Stephanie Zandee^5^, Chao Zhao^6^, Markus Kipp^7^, Kenneth J .Smith^8^, David Baker^4^, Daniel Altmann^9^, Stephen M. Anderton^5^, Yolanda S. Kap^10^, Jon D. Laman^10,11^, Bert A. ‘t Hart^10-12^, Moses Rodriguez^13^, Ralf Watzlawick^14^, Jan M. Schwab^15^, Roderick Carter^16^, Nicholas Morton^16^, Michele Zagnoni^17^, Robin J.M. Franklin^6^, Rory Mitchell^18^, Sue Fleetwood-Walker^18^, David A. Lyons^18^, Siddharthan Chandran^1,2^, Hans Lassmann^19^, Bruce D. Trapp^3^ and Don J. Mahad*^1^

^1^Centre for Clinical Brain Sciences, University of Edinburgh, Chancellor’s Building, 49 Little France Crescent, Edinburgh, EH16 4SB, UK

^2^UK Dementia Research Institute, University of Edinburgh, Edinburgh, UK

^3^Department of Neuroscience, Lerner Research Institute, Cleveland Clinic, Cleveland Ohio OH44195, USA

^4^Blizard Institute, Barts and The London School of Medicine and Dentistry, Queen Mary University of London, 4 Newark Street, London E1 2AT, UK

^5^Centre for Inflammation Research, University of Edinburgh, 47 Little France Crescent, Edinburgh, EH16 4SB, UK

^6^Wellcome Trust-MRC Cambridge Stem Cell Institute, Jeffrey Cheah Biomedical Centre, University of Cambridge, Cambridge Biomedical Campus, Cambridge CB2 0AW, UK

^7^Institute of Anatomy, Rostock University Medical Center, Gertrudenstrasse 9, 18057 Rostock, Germany

^8^Department of Neuroinflammation, The UCL Queen Square Institute of Neurology, University College London, 1 Wakefield Street, London, WC1N 1PJ, UK

^9^Faculty of Medicine, Department of Medicine, Hammersmith Campus, London, UK

^10^Department of Immunobiology, Biomedical Primate Research Centre, Rijswijk, The Netherlands

^11^Dept. Biomedical Sciences of Cells and Systems and MS Center Noord Nederland (MSCNN), University Groningen, University Medical Center Groningen, The Netherlands

^12^Dept. Anatomy and Neuroscience, Amsterdam University Medical Center (V|UMC|), Amsterdam, Netherlands

^13^Department of Neurology and Immunology, Mayo College of Medicine and Science, Rochester, Minnesota MN55905, USA

^14^ Department of Neurosurgery, Freiburg University Medical Center, Germany

^15^ Spinal Cord Injury Medicine, Department of Neurology, The Ohio State University, Wexner Medical Center, Columbus, USA

^16^Centre for Cardiovascular Science, Queens Medical Research Institute, 47 Little France Crescent, Edinburgh, UK

^17^Centre for Microsystems and Photonics, Electronic and Electrical Engineering, University of Strathclyde, Glasgow, UK

^18^Centre for Discovery Brain Science, Edinburgh Medical School, College of Medicine & Veterinary Medicine, University of Edinburgh, UK

^19^Department of Neuroimmunology, Center for Brain Research, Medical University Vienna, Spitalgasse 4, 1090, Vienna, Austria

**Key words:** multiple sclerosis, mitochondria, demyelination and neuroprotection

*Correspondence to: Dr D J Mahad, ^1^Centre for Clinical Brain Sciences, University of Edinburgh, Chancellor’s Building, 49 Little France Crescent, Edinburgh, EH16 4SB, UK

Email: don.mahad@ed.ac.uk Tel +44 131 2426164. Fax +44 191 2228553

†Joint first authors.

**Supplementary material**

Supplementary Figure 1


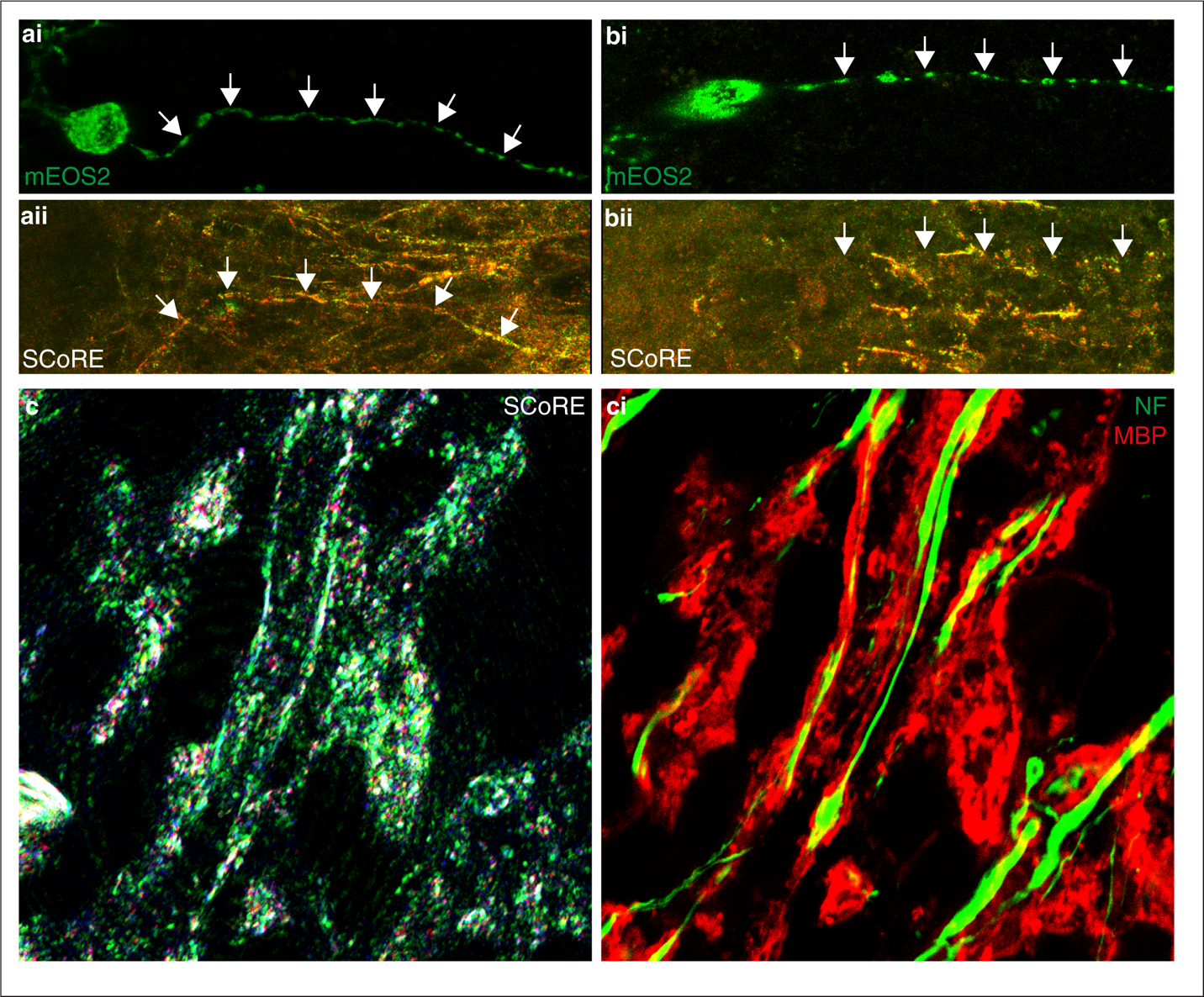


Supplementary Figure 1: Spectral Confocal Reflectance microscopy (SCoRe) can be used to determine the myelin status of Purkinje cell axons in cerebellar slices during live imaging. Mitochondrial fusion events are more abundant in demyelinated compared to myelinated Purkinje cell axons in cerebellar slices.

**a-b**: SCoRe images of myelinated (**aii**) and demyelinated (**bii**) axons were acquired in the same xy region and z-plane as the mEOS2 images (**ai** myelinated axon, **bi** demyelinated axon). SCoRe images showed positive signal along the mEOS2 positive myelinated axons, while demyelinated axons either showed a disrupted SCoRe signal or no SCoRe signal at all along the mEOS2 positive axons.

**c**: To confirm the specificity of SCoRe, cerebellar slices were fixed and stained using Immunofluorescence Histochemistry for NF and MBP to determine the overlap of SCoRe positive regions with the presence of myelinated or demyelinated mEOS2 positive axons.

Supplementary Figure 2


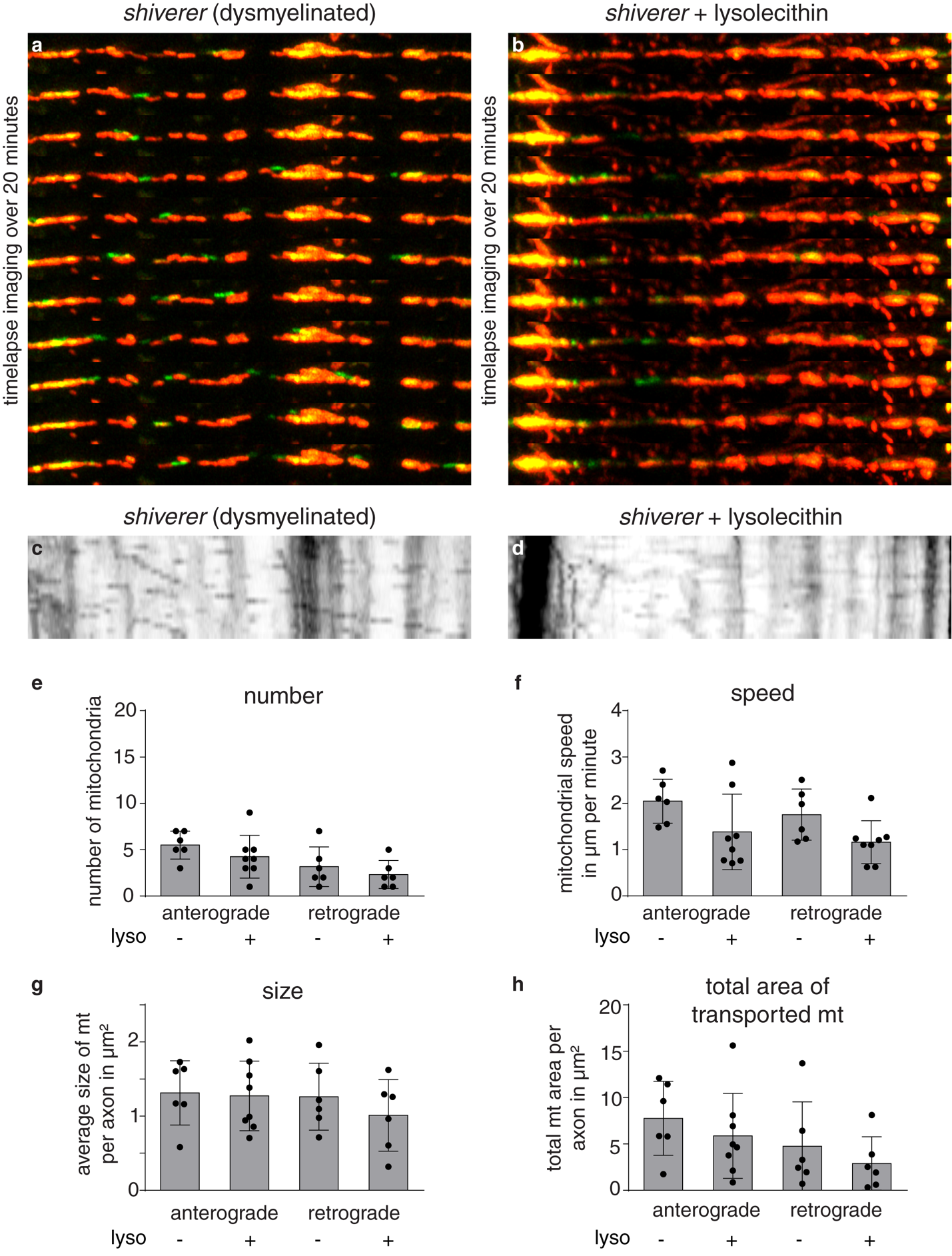


Supplementary Figure 2: Lysolecithin does not impact mitochondrial dynamics within dysmyelinated axon in *Shiverer* mice.

**a-d**: Time-lapse imaging of Purkinje cell mitochondria labeled with photoconvertible mEOS2 (green in unconverted state) in cerebellar slice cultures show the presence of newly transported mitochondria (green) within axons lacking myelin, or dysmyelinated axons, in *Shiverer* mice (**a**). Kymograph shows both anterograde (left to right) and retrograde movement of newly transported mitochondria within the dysmyelinated axons (**c**). Similar findings were noted when cerebellar slices from *Shiverer* mice were exposed to lysolecithin (**b** and **d**).

**e-h**: Quantitation of the newly transported mitochondria in the chronically dysmyelinated axons shows that lysolecithin does not significantly impact axonal mitochondrial dynamics. Mann-Whitney-U test for 2 group comparisons did not show a statistically significant change.

Supplementary Figure 3


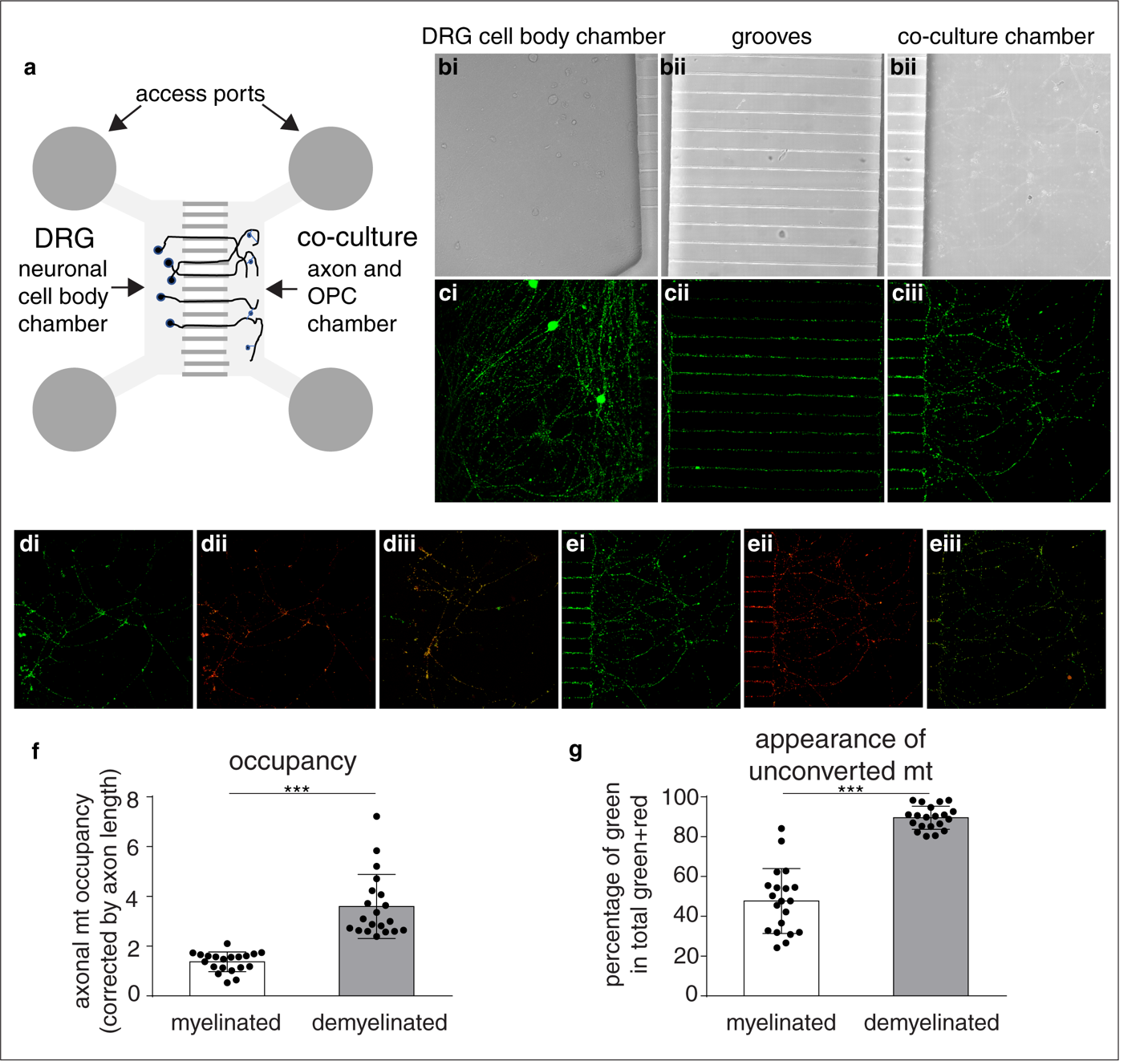


Supplementary Figure 3. Unconverted mitochondria more abundant in axons following demyelination in microfluidic chambers.

**a-c**: Application of lentivirus-mitochondria-targeted mEOS2 to dorsal root ganglia (DRG) neurons in the neuronal cell body compartment of microfluidic chambers (**a**, **bi** and **ci**) labels mitochondria in DRG neurons, including mitochondria within the axons that traverse the grooves between the chambers (**a**, **bii** and **cii**) and enter the co-culture chamber (**a**, **biii** and **ciii**). Oligodendrocyte progenitor cells (OPCs) in the co-culture chamber (**a**, **biii** and **ciii**) myelinate axons.

**d-e**: Photoconversion of mEOS2 labeled mitochondria in the co-culture chamber (**di** and **dii**) allows mitochondria that subsequently enter the photoconverted region to be assessed in myelinated axon segments (green in **diii**) as well as demyelinated axons [(green in **eiii** following photoconversion of green (**ei**) to red (**eii**)].

**f-g**: Lysolecithin-induced demyelination led to a significant increase in mitochondrial content within axons in the co-culture chamber, 16 hours post-lysolecithin exposure (**f**, when green and red channels are merged), as previously reported, *in vivo*^4^*.*Furthermore, the area of green labeled mitochondria in axons as a percentage of total area of mitochondria (when green and red channels are merged) as well as photoconverted mitochondria (red) is significantly greater following exposure to lysolecithin (**g**), indicating the greater movement of mitochondria from outside the photoconverted regions to the photoconverted region, following demyelination. Data presented as dot-plot with mean (bar) ± standard deviation (whiskers). ***p<0.001 using Mann-Whitney-U test.

Supplementary Figure 4


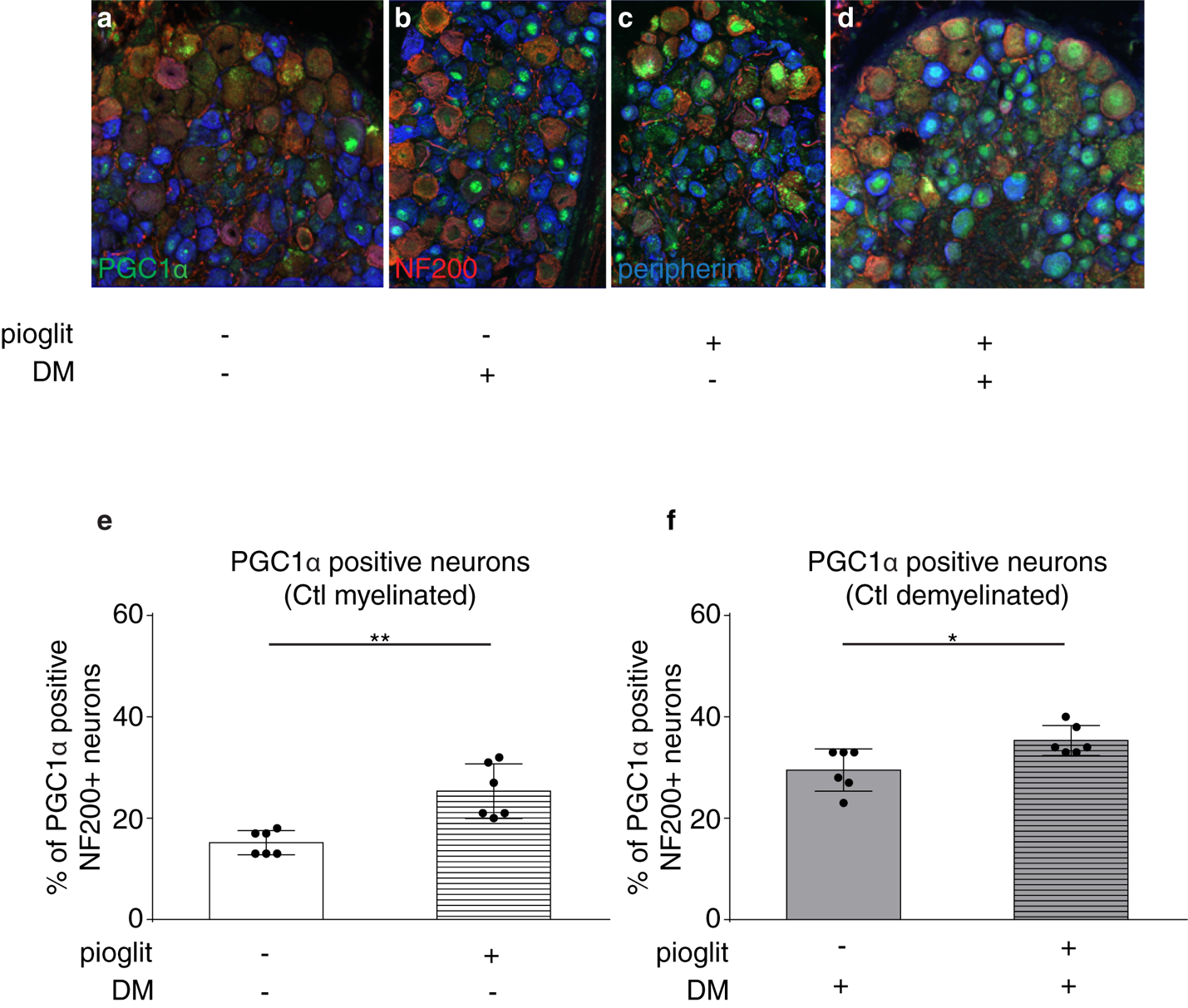


Supplementary Figure 4: PGC1α positive nuclei within DRG neurons in wild type mice increases significantly following focal demyelination of the dorsal columns and administration of pioglitazone in diet.

**a-f**: PGC1α positive neuronal nuclei (green) are relatively infrequent within DRG neurons from wild type mice on chow diet (**a**, untreated and not demyelinated). Pioglitazone in diet for 6 weeks significantly increases the percentage of DRG neurons with PGC1α positive nuclei (**c** and **e**) in wild type mice (neg) compared with mice on chow diet (**a** and **e**). Demyelination of the dorsal columns of untreated wild type mice (neg), using lysolecithin, increases in PGC1α positive DRG neuronal nuclei (**b**) compared with untreated and non-demyelinated wild type animals (**a**, **e** and **f**). Demyelination of the dorsal columns of pioglitazone treated wild type mice (neg), using lysolecithin, further increases in PGC1α positive DRG neuronal nuclei (**d** and **f**) compared with untreated and demyelinated wild type animals (**b** and **f**).

Data presented as dot-plot with mean (bar) ± standard deviation (whiskers). *p<0.05 and **p<0.01 using Mann-Whitney-U test.

Supplementary Figure 5


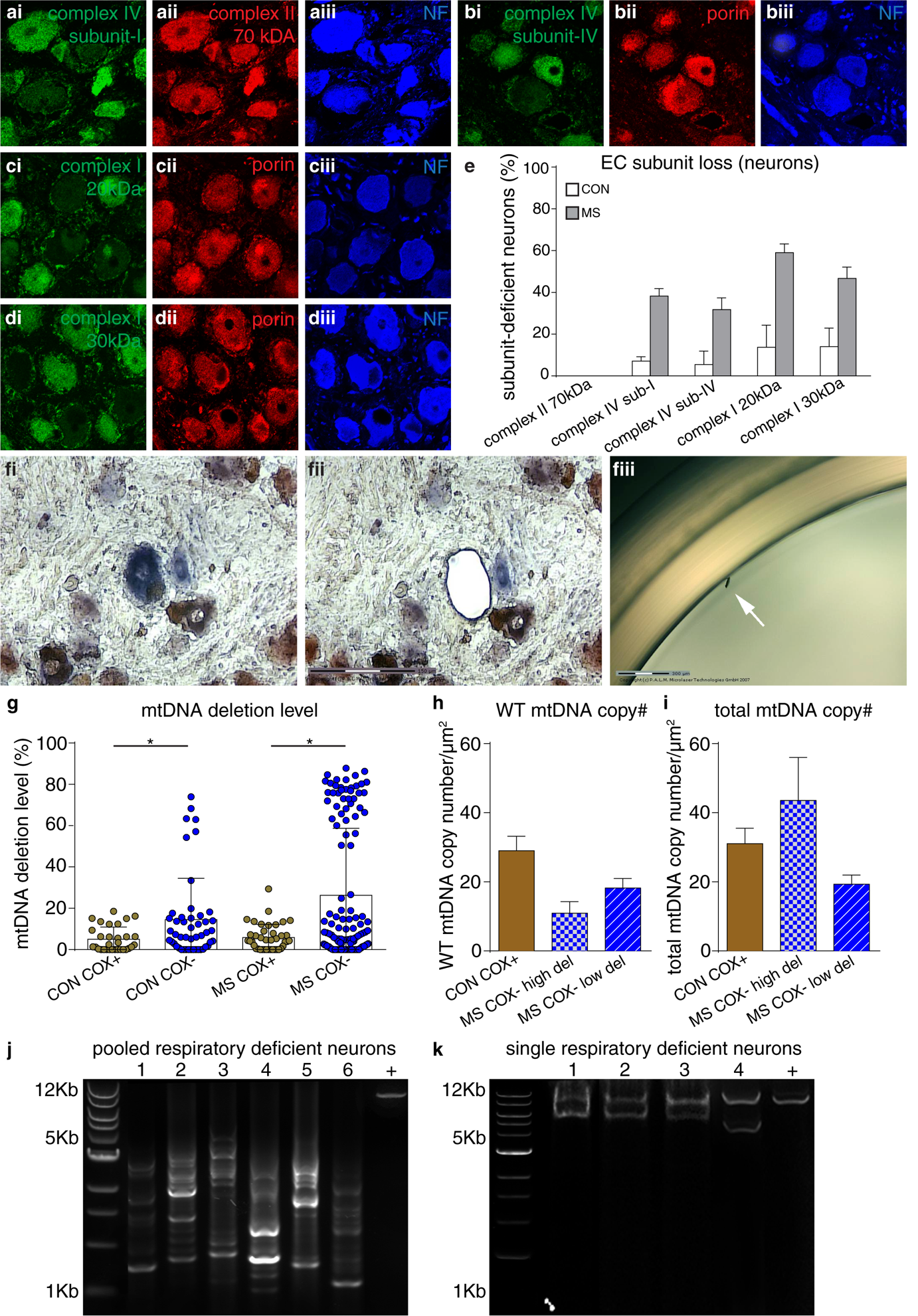


Supplementary Figure 5: Respiratory deficient DRG neurons in progressive MS lack mitochondrial respiratory chain complex subunits and harbor clonally expanded mitochondrial DNA deletions at a high heteroplasmy level.

**a-d**: Immunofluorescent labeling of mitochondrial respiratory chain complex subunits in DRG in progressive MS.

Complex IV subunit-I (**ai**, green), complex II 70kDa (**aii**, red) and total neurofilament (**aiii**, blue) triple labeling identified a subset of neurons with the complex II subunit and lacking the mitochondrial DNA encoded complex IV subunit-I in MS DRG (**a**, arrowheads).

Complex IV subunit-IV (**bi**, green), porin (**bii**, red, a voltage gated anion channel expressed on all mitochondria) and total neurofilament (**biii**, blue) triple labeling identified a subset of neurons lacking the above complex IV subunit in MS DRG (**b**, arrowheads). The complex IV subunit-IV labeling was detected in the peri-neuronal region surround the subunit deficient neurons.

Complex I 20kDa (**ci**, green), porin (**cii**, red) and total neurofilament (**ciii**, blue) triple labeling identified a subset of neurons lacking the above complex I subunit in MS DRG (**c**, arrowheads). As with the complex IV subunits, complex I 20kDa labeling was detected in the peri-neuronal region surround the subunit deficient neurons.

Complex I 30kDa (**di**, green), porin (**dii**, red) and total neurofilament (**diii**, blue) triple labeling identified a subset of neurons lacking the complex I subunit in MS DRG (**d**, arrowheads). As with the complex IV subunits and complex I 20kDa, complex I 30kDa labeling was detected in the peri-neuronal region surround the subunit deficient neurons.

**e**: Quantitation of DRG neurons lacking the subunits identified significantly greater percentage of neurons with loss of subunits in progressive MS than controls.

**f**: Respiratory deficient neurons (blue) and neurons with intact complex IV (brown) in cryostat sections placed on membrane slides (**fi**). Following laser microdissection (**fii**), the captured single neuron is apparent in the cap of sterile eppendorf tube (**fiii**, arrow).

**g**: When the level of mitochondrial DNA (mtDNA) deletion was determined at a single cell level by *MTND1/MTND4* real time PCR, the mean percentage of mtDNA deletion was significantly greater in respiratory deficient neurons (blue, COX negative) than those with intact complex IV activity (brown, COX positive) in both controls and multiple sclerosis (MS). The high level of mtDNA deletion (>50% threshold consistently reported) adequately explained the biochemical defect in 6 out of the 48 respiratory deficient neurons in control and 40 out of the 128 in MS. *p<0.05 using Mann-Whitney-U test.

**h-i**: As expected respiratory deficient neurons containing high heteroplasmy level of mtDNA deletion contained significantly less wild type mtDNA (**h**). The total mtDNA copy number did not significantly differ in respiratory deficient neurons from MS cases (MS COX negative) compared with neurons from control (CON, mostly consists of cells with intact complex IV activity). However, the respiratory deficient neurons harboring high level mtDNA deletion in MS contained significantly more total mtDNA copies (*MTND1*) than those with low level mtDNA deletion in MS as well as the neurons from controls (**i**).

**j-k**: To further characterize the mtDNA deletions in MS, long range PCR was performed on pooled respiratory deficient neurons (n=20 cells per lane), where multiple deletions of mtDNA were detected (**j**). In contrast to the multiple mtDNA deletions within pooled respiratory deficient DRG neurons, pooled neurons with intact complex IV activity (n=20 per lane) showed relatively few mtDNA deletions (not shown). When long range PCR was performed on single respiratory deficient neurons, mostly one mtDNA deletion was detected within single cells, which is consistent with clonal expansion of mtDNA deletion (**k**).

Data presented as dot-plot with mean (bar) ± standard deviation (whiskers).

Supplementary Figure 6


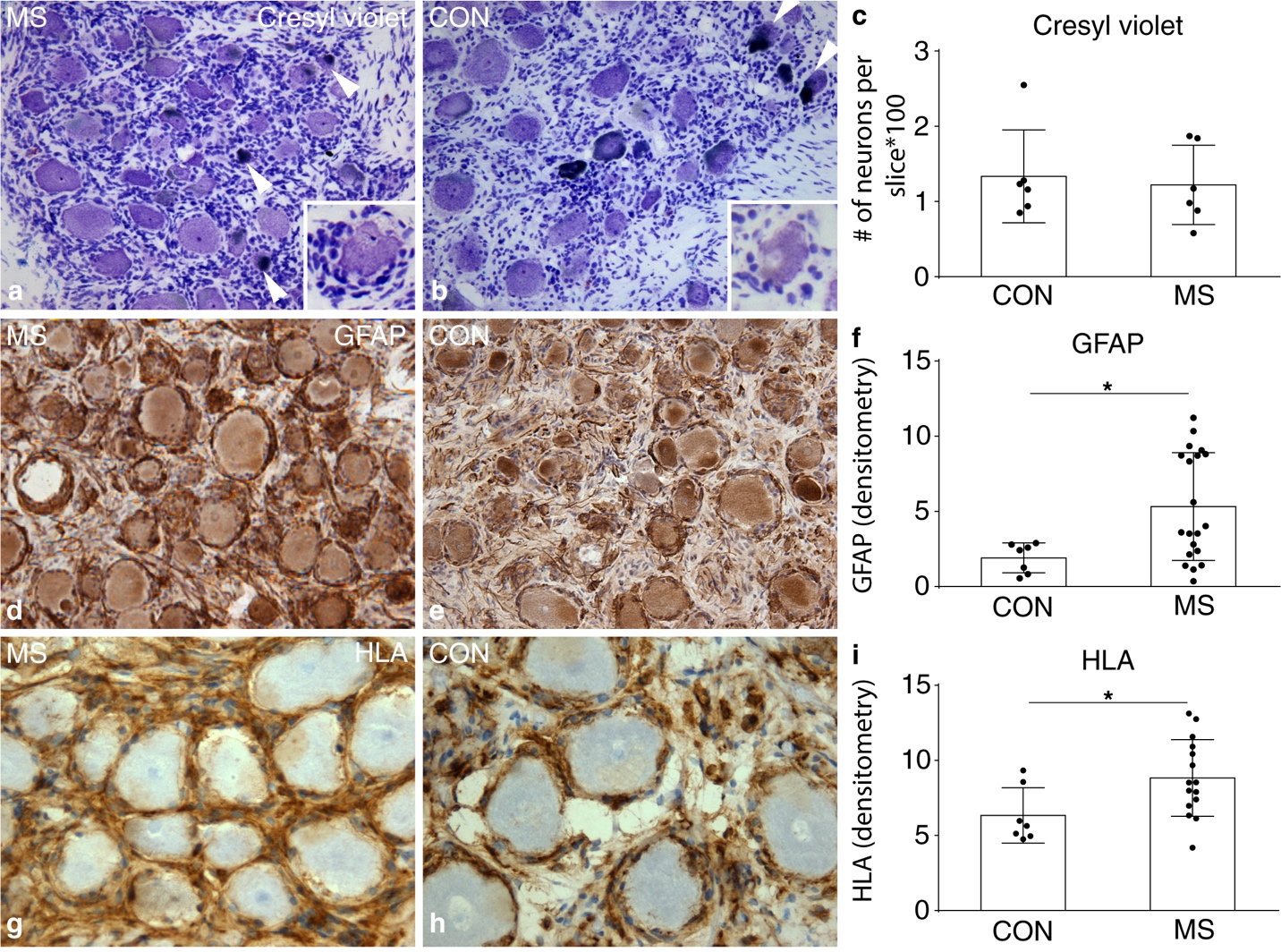


Supplementary Figure 6: Histological findings of dorsal root ganglia in progressive MS.

**a-c:** Cresyl violet staining of dorsal root ganglia (DRG) from progressive MS cases and controls show an increased cellularity of DRGs in MS (a and b). Quantitation of neuronal cell bodies did not identify a significant difference between the total number of DRG neurons in MS compared with control (c).

**d-f:** Immunohistochemical staining of DRG using antibodies against glial fibrillary acidic protein (GFAP) shows increased immunoreactivity in DRG of MS cases compared with control (d and e). Quantitation of GFAP staining using densitometry identifies a significant increase in GFAP immunoreactivity in DRG in MS compared with control (f).

**g-i:** Immunohistochemical staining of DRG using antibodies against Human leukocyte antigen (HLA) shows increased immunoreactivity in DRG of MS cases compared with control (g and h). Quantitation of HLA staining using densitometry identifies a significant increase in HLA immunoreactivity in DRG in MS compared with control (i).

Data presented as dot-plot with mean (bar) ± standard deviation (whiskers). *p<0.05, using Mann-Whitney-U test.

Supplementary Figure 7


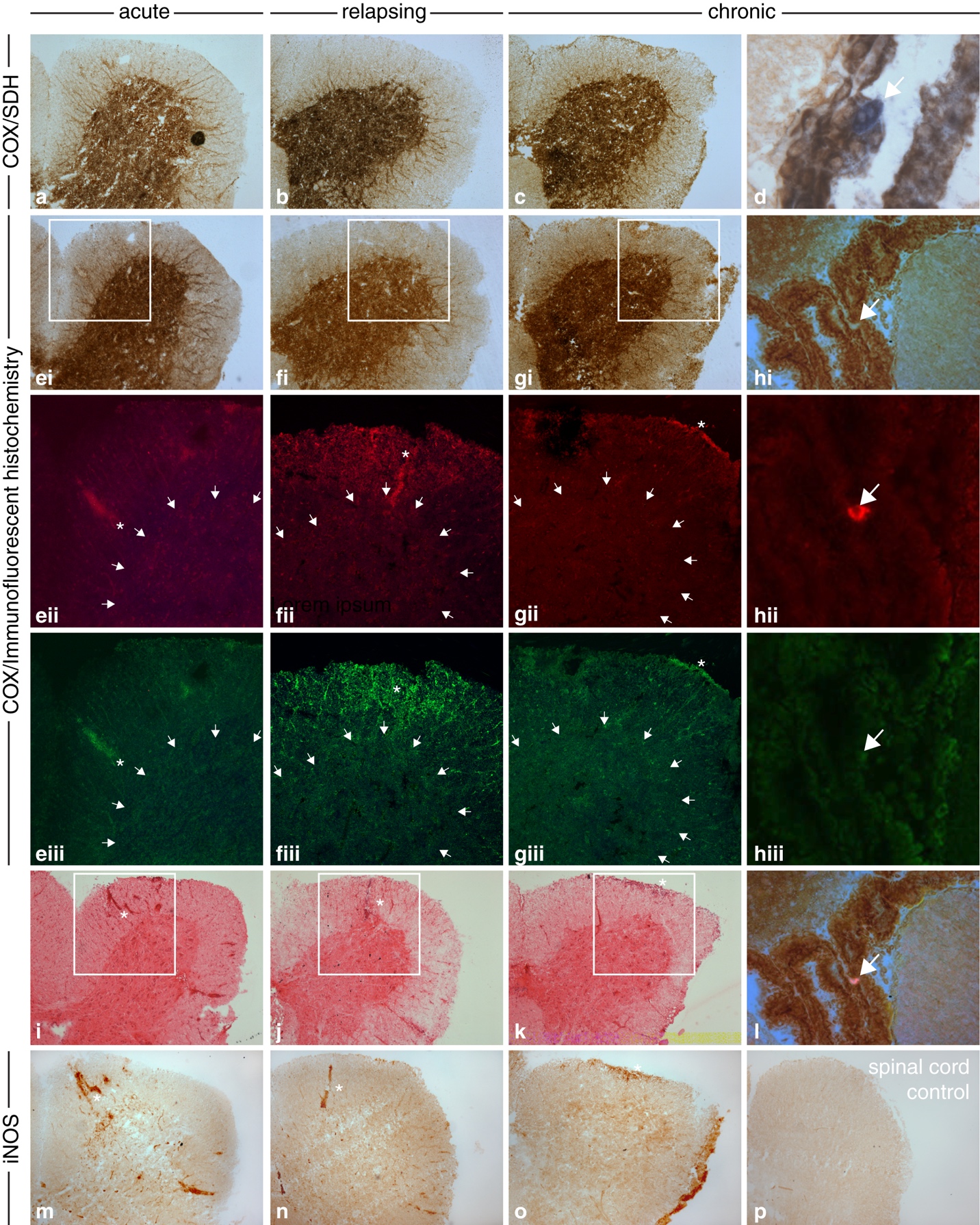


Supplementary Figure 7: Disease models lack respiratory deficient cells in the brain, spinal cord and dorsal root ganglia.

**a-c** illustrates the lack of cells deficient in complex IV and with intact complex II (respiratory-deficient, identified by sequential COX/SDH assay) in the spinal cord from Biozzi ABH EAE mice at acute (**a**), relapsing (**b**) and chronic stages (**c**).

**d-l**: Respiratory deficient choroid plexus epithelial cells that lack complex IV and contain complex II (**d**, stained blue, arrow) were infrequently found at the chronic stage of Biozzi ABH EAE mice (6 in 10 animals). The sequential COX histochemistry (**ei-gi**) and immunofluorescent labeling of complex II 70kDa (**eii-gii**, red) and complex IV subunit-I (**eiii**-**giii**, green) assay identified complex IV-deficient mitochondria, as previously reported, in spinal cord regions with inflammation in Biozzi ABH EAE. Complex VI deficient mitochondria contain both the subunits in the spinal cord (asterisks). The respiratory-deficient choroid plexus epithelial cells, however, contains complex II 70kDa (**hii**, arrowhead) and lack complex IV subunit-I (**hiii**, arrowhead), as reported in progressive MS. The merged image of complex IV activity (**hi**) and immunofluorescent labeling of subunits (**hii** and **hiii**) is shown in **l** (arrowhead showing the respiratory-deficient cells). The regions of the white matter that lacked complex IV activity despite the presence of complex IV subunit-I (indicated by asterisk in **e-g**) correspond to regions containing inflammatory infiltrates (asterisks) in acute (**i**), relapsing (**j**) and chronic phases (**k**), as shown by H&E staining of serial sections.

**m-p**: These regions with inflammation showed immunoreactivity for inducible nitric oxide synthase (iNOS) in acute (**m**), relapsing (**n**) and chronic phases (**o**) compared with control tissue (**p**).

Supplementary Figure 8


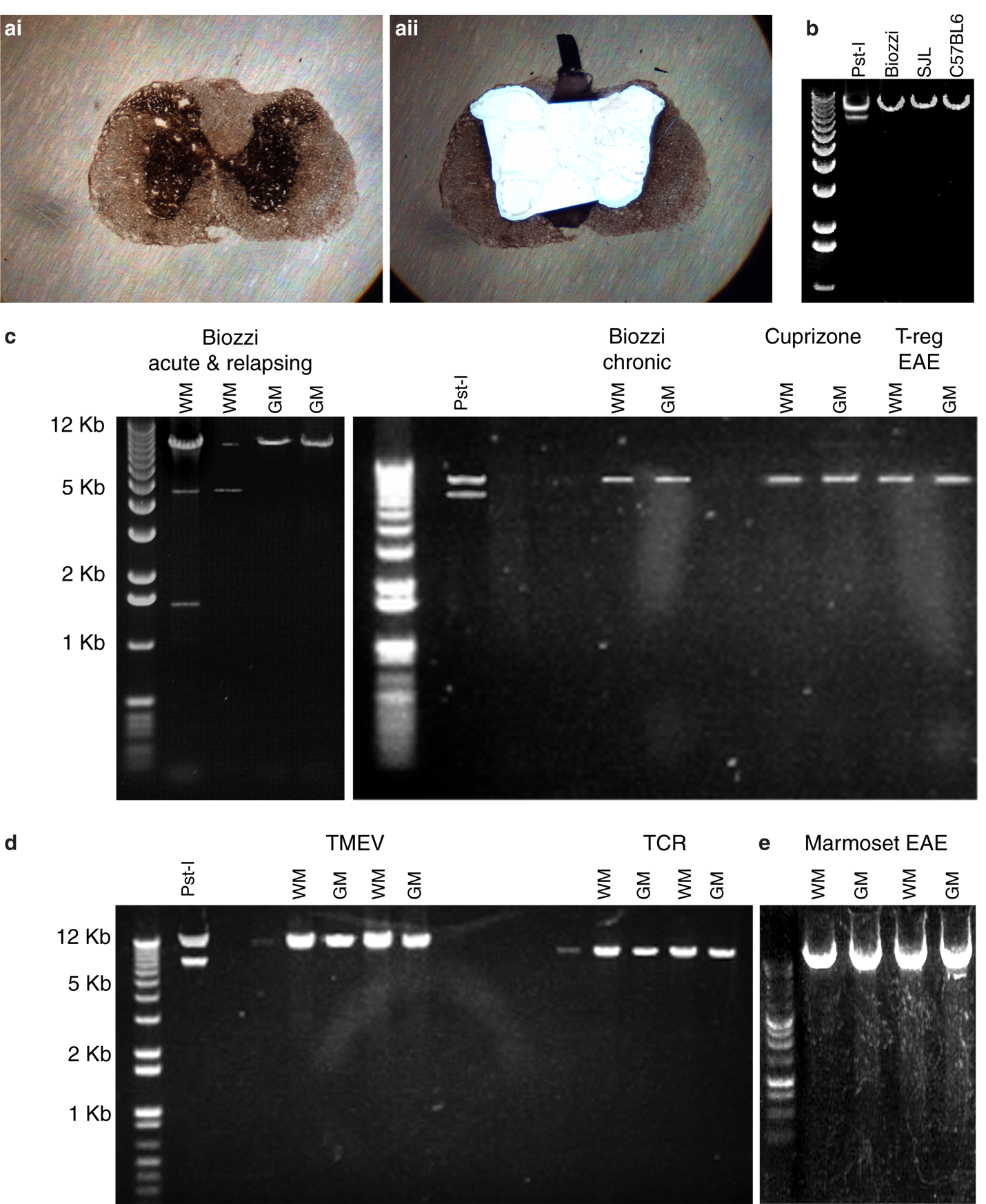


Supplementary Figure 8: Mitochondrial DNA deletions are rarely detected within the CNS in the disease models.

**a-b**: The spinal cord grey matter (GM) in cryosections with inflammation is microdissected, as evident in pre- and post- laser capture images (**a**). PstI tissue shows a mitochondrial DNA deletion (lower band), as a positive control (**b**). Only the wild type bands are seen in control Biozzi ABH, SJL and C57 mouse spinal cord grey matter (**b**).

**c-e**: Acute and relapsing phases of Biozzi EAE showed mtDNA deletions in (**c**, arrowheads) the white matter (WM), which were absent at the chronic stage and in the grey matter. Mitochondrial DNA deletions are not detected in the cuprizone model and T-reg depleted EAE (**c**) as well as Theiler’s murine encephalomyelitis (TMEV) and human T cell receptor (TCR) transgenic mice with spontaneous EAE (**d**) as well as marmoset EAE (**e**).

Supplementary Figure 9


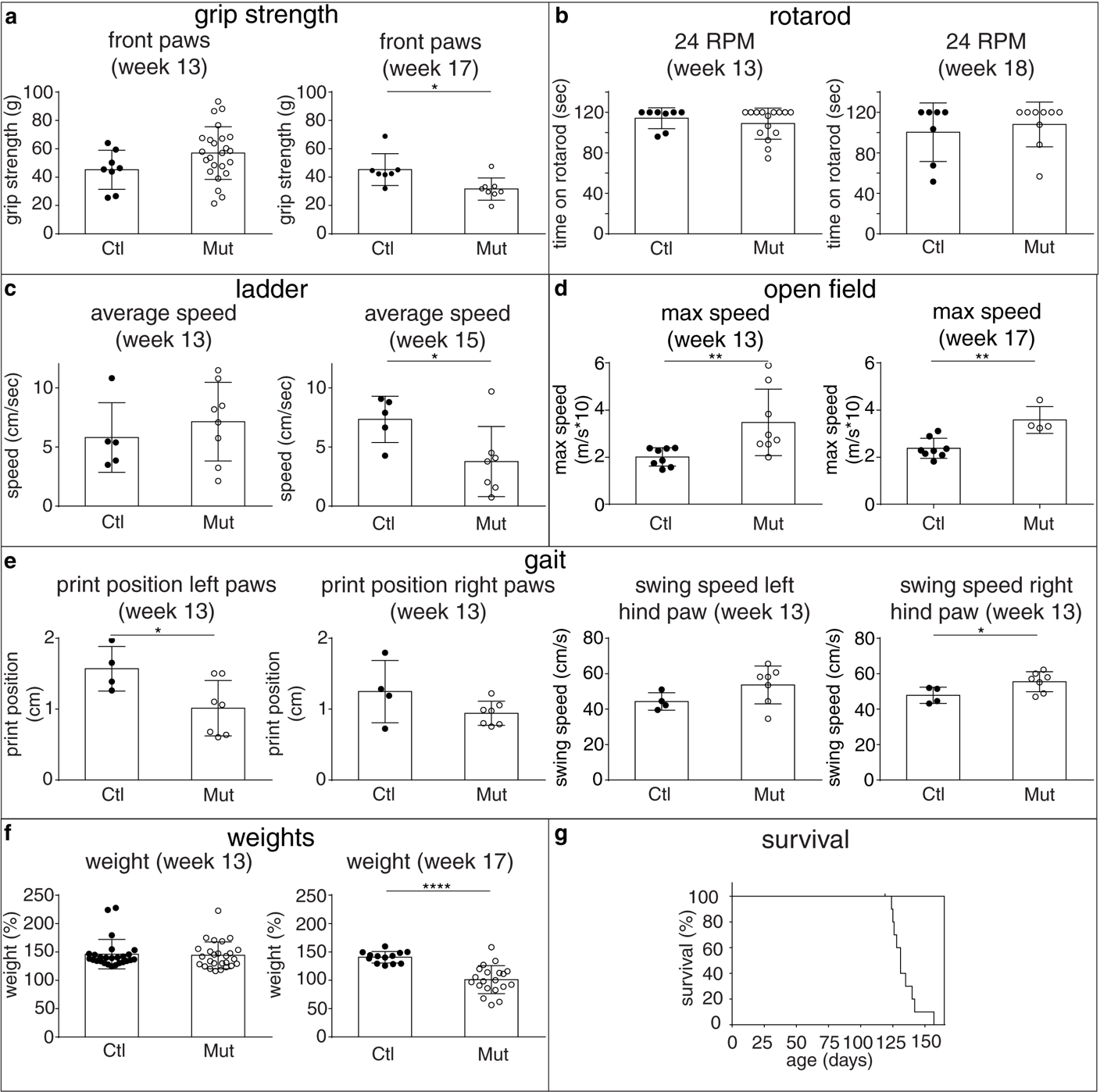


Supplementary Figure 9: Behavioral testing reveals a subtle clinical phenotype of COX10Adv mutant mice when experimental demyelination is carried out.

**a-d**: 13 weeks following the completion of tamoxifen gavaging, the time point at which focal experimental demyelination of the spinal cord was carried out, COX10Adv mutant mice do not show a significant difference in front paw grip strength (**a**), rotarod performance at 24 rpm (**b**), performance on horizontal ladder testing (**c**) and all but one parameter on open filed testing (**d**). Maximum speed on open field testing is significantly greater in the COX10Adv mutant mice compared with control mice (**d**).

**e**: Detailed gait analysis using CatWalk system shows a significantly lower print position of left paws and a significantly greater swing speed of right hind paw at 13 weeks following completion of tamoxifen in COX10Adv mutant mice compared with control mice, which are the only significant behavioral findings in COX10Adv mutant mice at 13 weeks post-tamoxifen, out of 20 parameters. At later time points, we detect significant changes in a number of behavioral parameters. Grip strength of front paws is significantly lower at 17 weeks (**a**) and average speed on horizontal ladder (**c**) is significantly lower at 15 weeks in COX10Adv mutant mice compared with controls.

**f-g**: The average weight of COX10Adv mutant mice is not significantly different compared with control mice at 13 weeks following completion of tamoxifen gavages, time point at which focal spinal cord demyelination was carried out. The survival of COX10Adv mutant mice, based on the development of a moderate clinical phenotype according to UK Home Office guidelines, is not compromised until 17 weeks following completion of tamoxifen gavaging.

Data presented as dot-plot with mean (bar) ± standard deviation (whiskers). *p<0.05, **p<0.01 and ****P<0.0001 using Mann-Whitney-U test and Student t-test.

Supplementary Figure 10

**
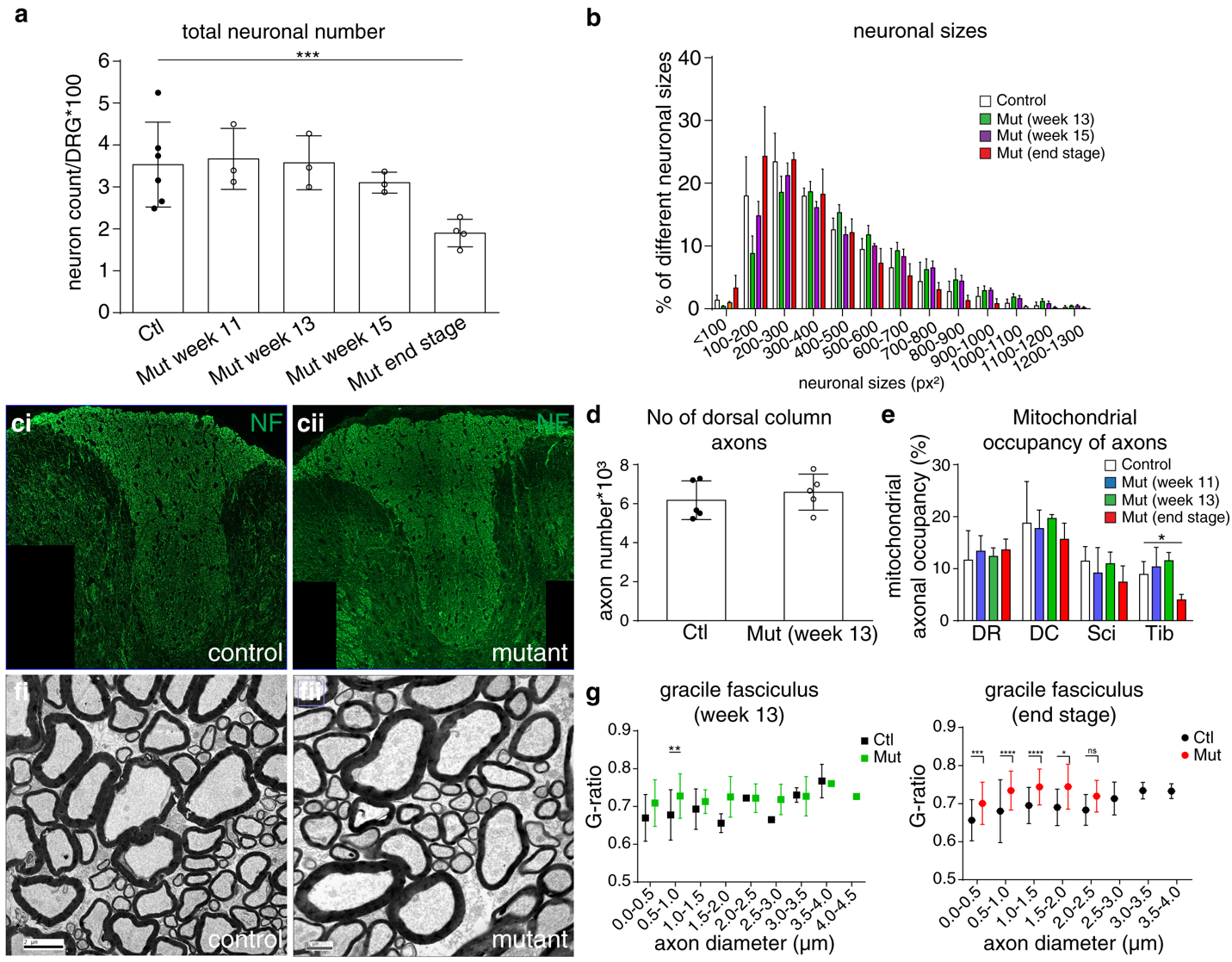
**

Supplementary Figure 10: No evidence of neurodegeneration at the time point when experimental demyelination is carried out.

**a-b**: The total number of neurons in the lumbar DRG, when counted in every 5 serial sections of the DRG, is not significantly different in COX10Adv mutant mice compared with control mice, at 13 weeks following the completion of tamoxifen gavages when experimental demyelination is carried out (**a**). Furthermore, the average area of DRG neurons in cresyl violet staining indicates the preservation of relatively large neurons at the time point when demyelination is carried out (**b**).

**c-d**: In cross-sections of the thoracic spinal cord, the total number of dorsal column axons are not significantly different in COX10Adv mutant mice (**cii** and **d**) compared with control mice (**ci** and **d**).

**e**: Mitochondrial content of myelinated axons, as a percentage of the area of the axon occupied by mitochondria, is not significantly different at the time point when experimental demyelination is carried out. In contrast, a significant depletion of mitochondria is apparent within myelinated axons in sciatic nerve and tibial nerve at the end stage of COX10Adv mutant mice compared with control mice, as expected^21^.

**f-g**: The average g-ratio of dorsal column axons (gracile fasciculus) in COX10Adv mutant mice is not significantly different from control mice at 13 weeks following the completion of tamoxifen gavages when experimental demyelination is carried out (**g**). In contrast, the average g-ratios are significantly greater in COX10Adv mutant mice at the end stage compared with control mice, indicating potentially thinner myelin (**g**). Degenerating axons are absent in both COX10Adv mutant mice (**fii**) and control mice (**fi**) at 13 weeks following the completion of tamoxifen gavages when experimental demyelination is carried out.

Data presented as dot-plot with mean (bar) ± standard deviation (whiskers). *p<0.05, **p<0.01, ***p<0.001 and ****P<0.0001 using Mann-Whitney-U test and Kruskal-Wallis test showed a p<0.05 in multiple subgroup comparisons.

Supplementary Figure 11


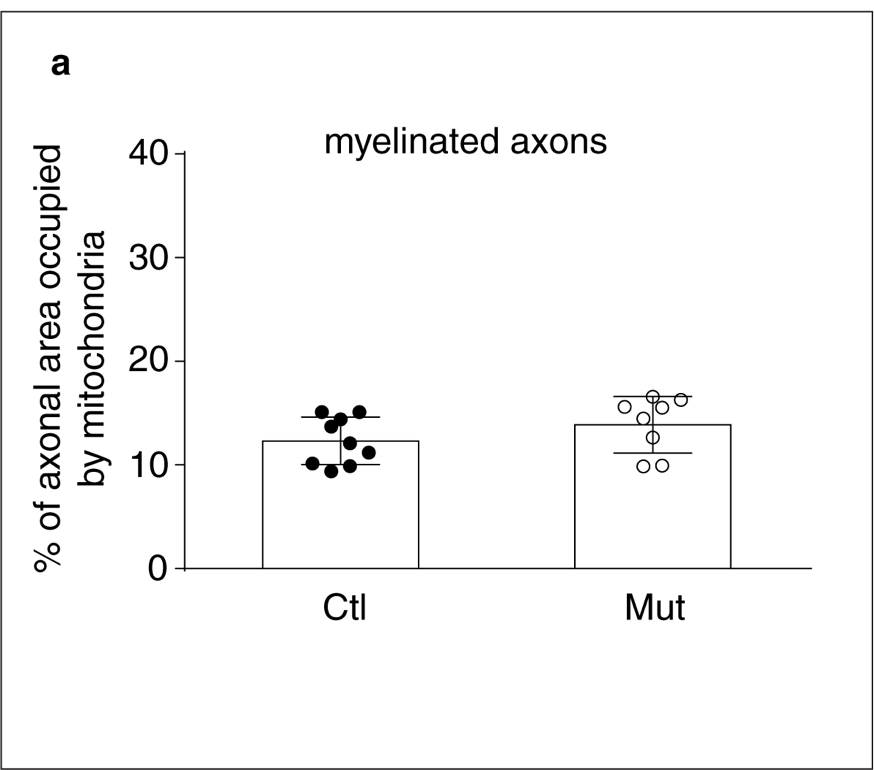


Supplementary Figure 11: Mitochondrial content within myelinated axons in COX10Adv mutant mice and controls.

**a**: Quantitation of axonal mitochondrial occupancy in myelinated axons of Wildtype and COX10Adv mutant mice did not find a significant difference of axonal mitochondrial occupancy in myelinated axons between COX10Adv mutant mice and wildtype controls and (p=0.228).

Supplementary Figure 12


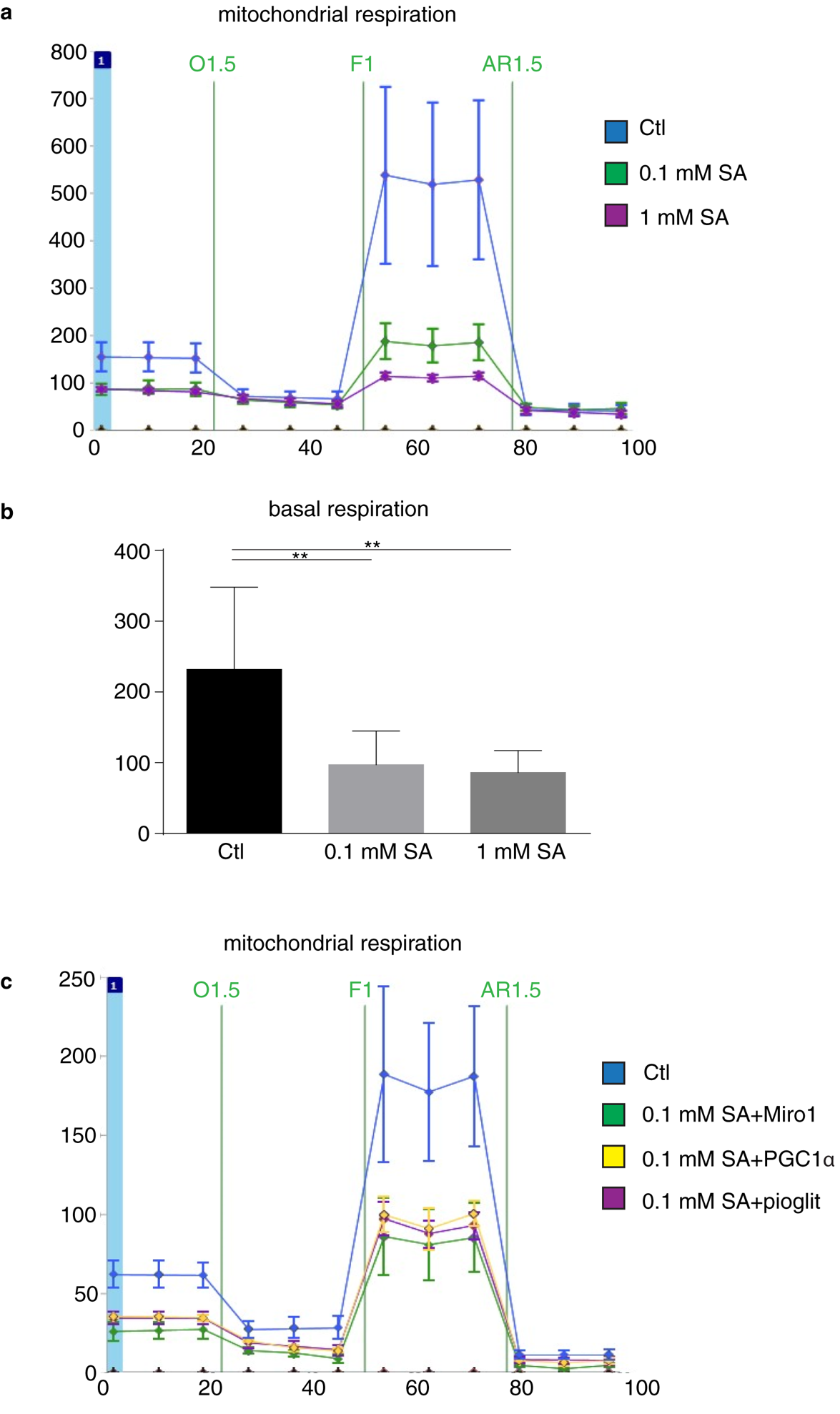


Supplementary Figure 12: Modeling the complex IV deficiency in DRG neurons, *in vitro*.

**a-c**: Application of sodium azide [SA (100μM for 16 hours)] to DRG neurons, *in vitro*, significantly decreased mitochondrial respiration in SeaHorse analysis (**a-b**), as expected, without compromising cell viability (not shown). Mitochondrial respiration decreases when DRG neurons that are over-expressing Miro1, over-expressing PGC1α or treated with pioglitazone are exposed to SA (100μM for 16 hours) (**c**). **p<0.01 using Mann-Whitney-U test once Kruskal-Wallis test showed a p<0.05.

Supplementary Figure 13


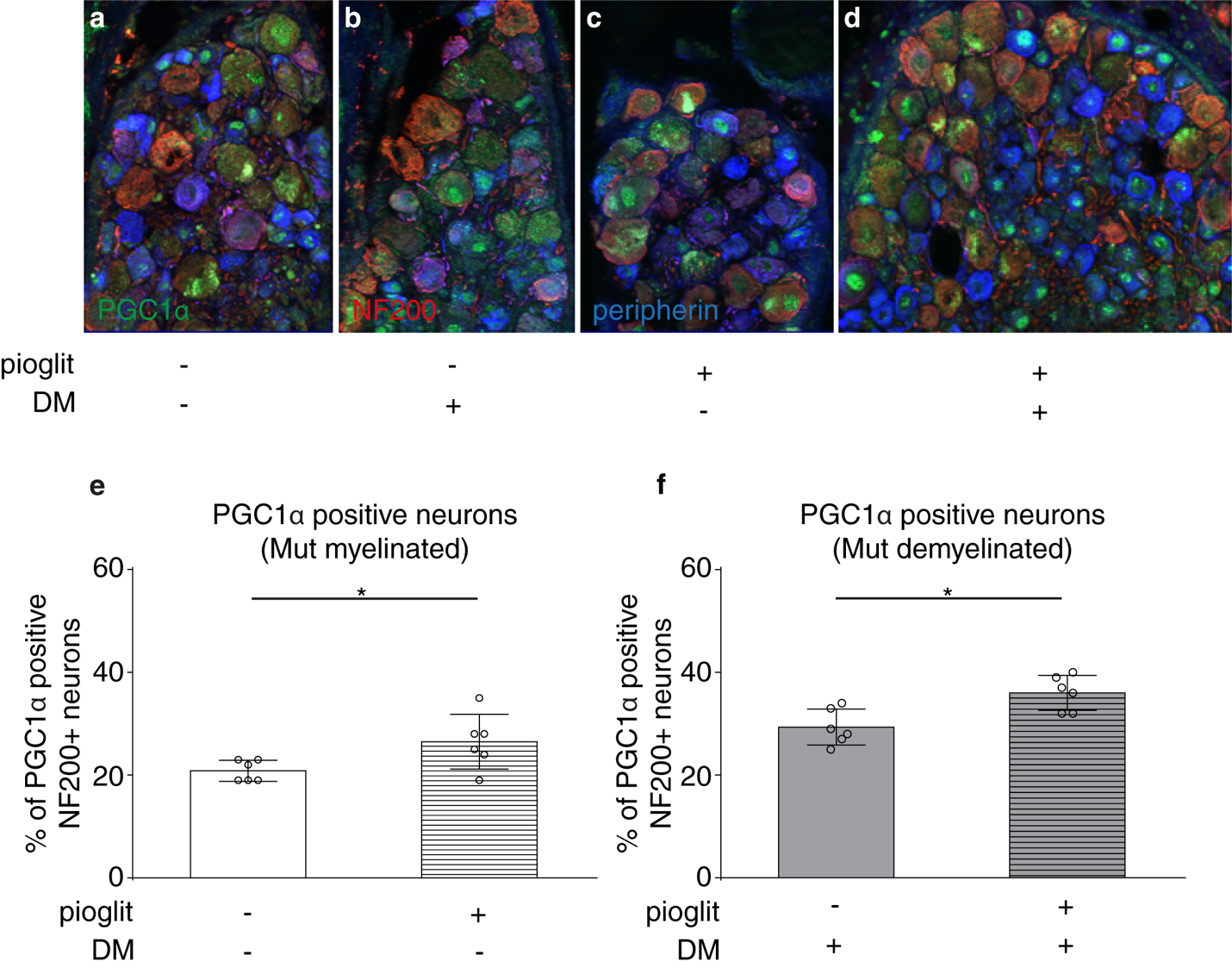


Supplementary Figure 13: PGC1α positive nuclei within DRG neurons in COX10Adv mutant mice increases significantly following focal demyelination of the dorsal columns and administration of pioglitazone in diet.

**a-f**: PGC1α positive neuronal nuclei (green) are relatively infrequent within DRG neurons from COX10Adv mutant mice on chow diet (**a**, untreated and not demyelinated). Pioglitazone in diet for 6 weeks significantly increases the percentage of DRG neurons with PGC1α positive nuclei (**c** and **e**) in COX10Adv mutant mice (neg) compared with COX10Adv mutant mice on chow diet (**a** and **e**). Demyelination of the dorsal columns of untreated COX10Adv mutant mice (neg), using lysolecithin, increases in PGC1α positive DRG neuronal nuclei (**b**) compared with untreated and non-demyelinated COX10Adv mutant mice (**a**, **e** and **f**). Demyelination of the dorsal columns of pioglitazone treated COX10Adv mutant mice (neg), using lysolecithin, further increases in PGC1α positive DRG neuronal nuclei (**d** and **f**) compared with untreated and demyelinated COX10Adv mutant mice (**b** and **f**).

Data presented as dot-plot with mean (bar) ± standard deviation (whiskers). *p<0.05 using Mann-Whitney-U test.

Supplementary Figure 14


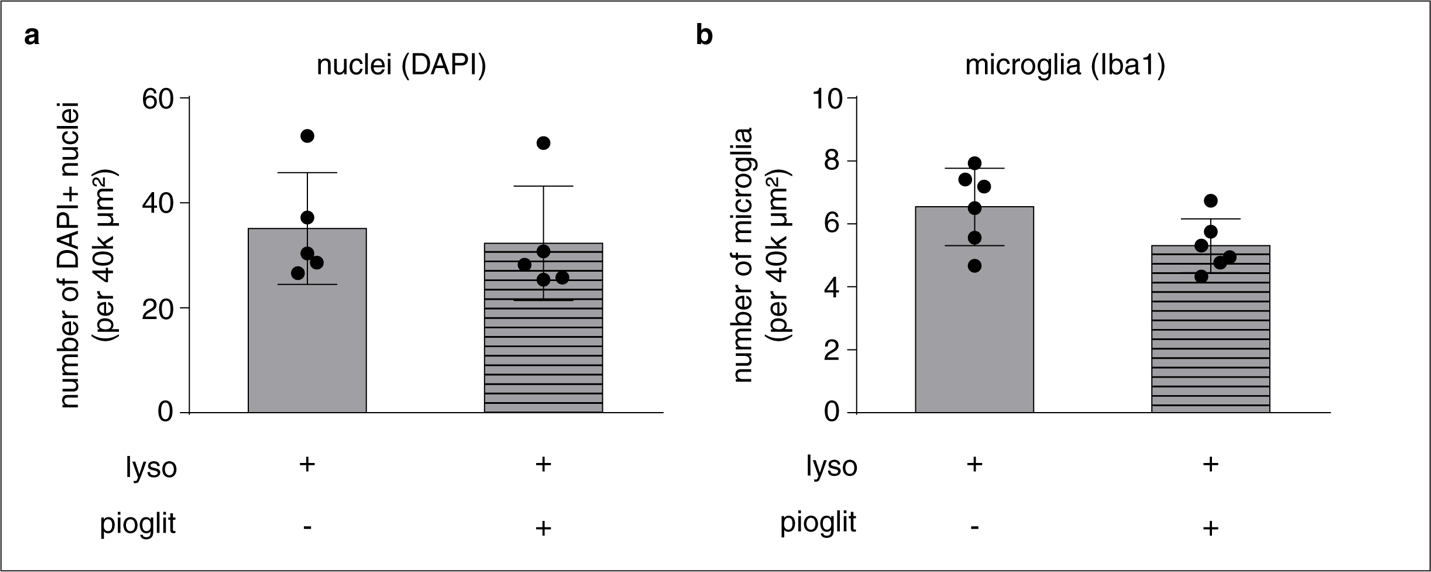


Supplementary Figure 14: Quantitation of DAPI+ nuclei and Iba1+ microglia in focal demyelinating lesions of the spinal cord dorsal columns in wild type mice, with and without Pioglitazone treatment.

**a-b**: Both the number of DAPI+ nuclei (**a**, p=0.413) and the number of Iba1+ microglia (**b**, p=0.132) in focal demyelinating lesions are not significantly different in Pioglitazone treatment mice compared with controls without Pioglitazone. However, there was a trend towards a decrease in microglial number within focal demyelinating lesions of Pioglitazone treated mice (**b**).

Supplementary Table 1

Details of antibodies used for immunohistochemistry and immunofluorescent histochemistry.

| **Antigen** | **Target** | **Antibody type** | **Source** |
| --- | --- | --- | --- |
| NF-L | Neurofilament light | Chicken polyclonal | EnCor |
| NF200 | Neurofilament heavy | Rabbit polyclonal | Sigma |
| SMI31 | Phosphorylated neurofilaments | Mouse IgG_1_ | Covance |
| SMI32 | Non-phosphorylated neurofilaments | Mouse IgG_1_ | Covance |
| MBP | Myelin basic protein | Rat polyclonal | Covance |
| MBP | Myelin basic protein | Rabbit polyclonal | Gift from Peter Brophy |
| COX-I | COX subunit-I | Mouse IgG_2a_ | Abcam |
| COX-IV | COX subunit-IV | Mouse IgG_2a_ | Abcam |
| SDHA II | SDH 70 kDa subunit | Mouse IgG_1_ | Abcam |
| Complex I 20 kDa subunit | NDUFS7 protein | Mouse IgG_1_ | Abcam |
| Complex I 30 kDa subunit | NDUFS3 protein | Mouse IgG_1_ | Abcam |
| Porin | Mitochondrial transmembrane protein | Mouse IgG_2b_ | Abcam |
| PGC1α | Peroxisome proliferator-activated receptor gamma coactivator 1-alpha | Rabbit polyclonal | Abcam |
| Peripherin | Peripherin | Mouse IgG_1_ | Sigma |
| Iba1 | Ionised calcium binding adaptor molecule 1 | Rabbit polyclonal | MenaPath |

COX: complex IV or cytochrome *c* oxidase. SDH: complex II or succinate dehydrogenase.
